# Luminescence and Electrochemical Activity of New Unsymmetrical 3-Imino-1,8-naphthalimide Derivatives

**DOI:** 10.3390/ma14195504

**Published:** 2021-09-23

**Authors:** Sonia Kotowicz, Mateusz Korzec, Katarzyna Malarz, Aleksandra Krystkowska, Anna Mrozek-Wilczkiewicz, Sylwia Golba, Mariola Siwy, Sebastian Maćkowski, Ewa Schab-Balcerzak

**Affiliations:** 1 Institute of Chemistry, University of Silesia, 9 Szkolna Str., 40-006 Katowice, Poland; ewa.schab-balcerzak@us.edu.pl; 2 A. Chelkowski Institute of Physics, University of Silesia in Katowice, 1A 75 Pulku Piechoty Str., 41-500 Chorzow, Poland; katarzyna.malarz@us.edu.pl (K.M.); aleksandra.krystkowska@us.edu.pl (A.K.); anna.mrozek-wilczkiewicz@us.edu.pl (A.M.-W.); 3 Institute of Materials Science, University of Silesia, 1A 75 Pulku Piechoty Str., 41-500 Chorzow, Poland; sylwia.golba@us.edu.pl; 4 Centre of Polymer and Carbon Materials, Polish Academy of Sciences, 34 M. Curie-Sklodowska Str., 41-819 Zabrze, Poland; msiwy@cmpw-pan.edu.pl; 5 Institute of Physics, Faculty of Physics, Astronomy and Informatics, Nicolaus Copernicus University, 5 Grudziadzka Str., 87-100 Torun, Poland; mackowski@fizyka.umk.pl

**Keywords:** 1,8-naphthalimides, electrochemistry, luminescence, imines, cell imaging

## Abstract

A new series of 1,8-naphtalimides containing an imine bond at the 3-position of the naphthalene ring was synthesized using ^1^H, ^13^C NMR, FTIR, and elementary analysis. The impact of the substituent in the imine linkage on the selected properties and bioimaging of the synthesized compounds was studied. They showed a melting temperature in the range of 120–164 °C and underwent thermal decomposition above 280 °C. Based on cyclic and differential pulse voltammetry, the electrochemical behavior of 1,8-naphtalimide derivatives was evaluated. The electrochemical reduction and oxidation processes were observed. The compounds were characterized by a low energy band gap (below 2.60 eV). Their photoluminescence activities were investigated in solution considering the solvent effect, in the aggregated and thin film, and a mixture of poly(*N*-vinylcarbazole) (PVK) and 2-tert-butylphenyl-5-biphenyl-1,3,4-oxadiazole (PBD) (50:50 wt.%). They demonstrated low emissions due to photoinduced electron transport (PET) occurring in the solution and aggregation, which caused photoluminescence quenching. Some of them exhibited light emission as thin films. They emitted light in the range of 495 to 535 nm, with photoluminescence quantum yield at 4%. Despite the significant overlapping of its absorption range with emission of the PVK:PBD, incomplete Förster energy transfer from the matrix to the luminophore was found. Moreover, its luminescence ability induced by external voltage was tested in the diode with guest–host configuration. The possibility of compound hydrolysis due to the presence of the imine bond was also discussed, which could be of importance in biological studies that evaluate 3-imino-1,8-naphatalimides as imaging tools and fluorescent materials for diagnostic applications and molecular bioimaging.

## 1. Introduction

1,8-Naphthalimide is a motif in compounds with a wide range of applications, for example, organic electronics (e.g., organic light emitting diodes (OLEDs), bulk heterojunction solar cells (BHJs), dye-sensitized solar cells (DSSCs), etc.) and cellular imaging; it also exhibits a broad spectrum of biological activity [1,2,3,4]. The wide range of applications is mainly considered in compounds substituted in the 4-C position of the naphthalene ring, which may be associated with obtaining a suitable donor-acceptor system. There is relatively little information about the optical or electronic properties of compounds with substituents in the 3-C position of the naphthalene ring. Moreover, despite the great interest in 4-C substituted compounds, only a few examples of imine derivatives can be found in the literature [5,6,7]. However, other analogues containing a 1,8-naphthalimide unit and electroluminescence ability were described [8]. Compounds used in OLEDs included, for example, vinyl derivatives [9], Schiff base moiety [10], or pyrene substituents [11]. The 1,8-naphthalimide derivatives were also described in the literature for cellular imaging [1,2]. They include various mechanisms responsible for their use, such as photoinduced electron transport inhibition [12], compound hydrolysis [13], aggregation-induced emission (AIE) [2,14], bioreductive fluorescent sensors [15], fluorogenic substrates for glutathione S-transferase (GST) activity [16], or the formation of metal complexes, i.e., Zn^2+^ [17,18,19], Cu^2+^ [20,21], Fe^3+^ [22,23]. Nevertheless, the use of imines in biological research is associated with the possibility of hydrolysis of these compounds [24]. However, the imines described in the literature, which underwent hydrolysis and had a significant impact on the properties, most often contained a unit with an imine bond in their structures, capable of complexing ions [25,26,27,28,29,30,31,32]. It should be noted that earlier studies have shown that, despite partial hydrolysis of the compounds, biological activity was associated with the presence of non-hydrolyzed imine [26]. Accordingly, the partial hydrolysis susceptibility of these compounds should be taken into account. In research, it should be determined whether this phenomenon constitutes a significant contribution to the described properties. 

In our previous works, for the first time, our group described properties of the 3-C substituted 1,8-naphthalimides containing an imine and β-ketoamine bond, and their applicability in OLED devices and cell imaging [28,33,34,35,36]. The 1,8-naphthalimide derivatives described in our publications were used for cellular imaging, including salicyl imine analogues undergoing hydrolysis [28] or were susceptible to aggregation-induced emission (the β-ketoenamines) [34,35]. In turn, the use of iminonaphthalimides in organic electronics included the structures of mono- and bis-iminonaphthalimides [33,35,36]. Most of the compounds tested showed the ability to electroluminescence, which is the first time our research showed this. It should be emphasized that there is no information on other 3- or 4-substitute imine 1,8-naphtalimides that would exhibit electroluminescent properties.

In this paper, we describe structural characteristics and thermal, electrochemical, optical, electroluminescent, and biological properties of six new azomethine 1,8-naphthalimide derivatives (AzNI). The spectroscopic tests for the analyzed compounds were performed to determine the properties in the aggregated state and the susceptibility of the compounds to the inhibition of the PET process. The influence of the partial imine hydrolysis, mainly due to its importance in our previous research [25,26,28], was also analyzed in this paper, from the point of view of its application, such as for cell imaging. In addition, the electroluminescent properties of the described compounds were tested and compared with the results obtained in previous studies, in which the structure of the device was similar. 

## 2. Experimental Section

### 2.1. Materials and Characterization Methods

All used materials are commercially available. The 3-Amino-*N*-hexyl-1,8-naphthalimide was prepared as described in [33,34]. The blends, films, OLED device preparations, and characterization methods are available in the Supporting Information (SI). 

### 2.2. Synthesis of N-Hexyl-1,8-naphthalimides Derivatives

The 3-Amino-*N*-hexyl-1,8-naphthalimide (1 mmol, 0.296 g) was dissolved in 6 cm^3^ of methanol and stirred. Aldehyde (1 eq. of: β-phenylcinnamaldehyde, 9-phenanthrenecarboxaldehyde, 4-bromo-benzaldehyde, 2,4-dichlorobenzaldehyde, p-nitrobenzaldehyde, 4-(1*H*-imidazol-1-yl)benzaldehyde)) was added to the dissolved 3-Amino-*N*-hexyl-1,8-naphthalimide. The condensation reaction was carried out for 24 h at 25 °C. Afterward, the product was filtered, washed with 20 cm^3^ of methanol, and dried in a vacuum oven at 60 °C for 6 h. 

3-(3,3-diphenylprop-2-en-1-yl)-*N*-hexyl-1,8-naphthalimide (AzNI-1)

Yellow solid. Yield = 26%. m = 0.12 g. ^1^H NMR (400 MHz, DMSO-*d*_6_, δ, ppm): 8.45–8.34 (m, 2H, -CH); 8.20 (d, *J* = 9.3 Hz, 1H, -CH=N-); 8.09 (s, 1H, -CH); 8.05 (s, 1H, -CH); 7.83 (t, *J* = 7.6 Hz, 1H, -CH); 7.55–7.48 (m, 3H, -CH); 7.45 (s, 5H, -CH); 7.38 (d, *J* = 7.6 Hz, 2H, -CH); 7.16 (d, *J* = 9.3 Hz, 1H, -CH=C-); 4.10–3.92 (m, 2H, -N-CH_2_-); 1.69–1.51 (m, 2H, -CH_2_-); 1.45–1.17 (m, 6H, -CH_2_-); 0.87 (t, *J* = 6.3 Hz, 3H, -CH_3_). ^13^C NMR (101 MHz, DMSO-*d*_6_, δ, ppm): 163.8; 163.6; 162.0; 155.6; 150.9; 140.6; 138.0; 134.5; 132.9; 130.7; 130.2; 130.0; 129.3; 129.2; 129.1; 128.4; 128.1; 127.0; 126.2; 125.0; 124.2; 123.7; 122.6; 31.3; 27.9; 26.6; 22.3; 14.2. FTIR (KBr, *v*, cm^−1^): 3058 (C-H aromatic); 2956, 2928 (C-H aliphatic); 1702, 1660 (C=O imide); 1626 (-CH=N- imine). Anal. Calcd. for C_32_H_28_N_2_O_2_ (472.58 g/mol): C (81.33%) H (5.97%) N (5.93%); found: C (81.40%) H (5.82%) N (5.89%).

3-(9-phenanthrene)-*N*-hexyl-1,8-naphthalimide (AzNI-2)

Yellow solid. Yield = 46%. m = 0.22 g. ^1^H NMR (400 MHz, DMSO-*d*_6_, δ, ppm): 9.62–9.49 (m, 1H); 9.47 (s, 1H); 9.01–8.98 (m, 1H); 8.92 (d, *J* = 8.4 Hz, 1H); 8.66 (s, 1H); 8.54 (d, *J* = 1.7 Hz, 4H); 8.47 (dd, *J*_1_ = 10.8 Hz, *J*_2_ = 8.6 Hz, 3H); 8.18 (d, *J* = 7.7 Hz, 1H); 7.97–7.79 (m, 4H); 7.76 (t, *J* = 7.3 Hz, 1H); 4.15–4.02 (m, 2H); 1.74–1.59 (m, 2H); 1.44–1.25 (m, 6H); 0.89 (t, *J* = 6.6 Hz, 3H). ^13^C NMR (101 MHz, DMSO-*d*_6_, δ, ppm): 163.9; 163.8; 163.7; 150.9; 134.8; 134.7; 133.1; 131.8; 130.9; 130.8; 130.4; 130.0; 129.9; 129.6; 129.5; 128.4; 128.1; 128.0; 127.9; 126.3; 126.1; 124.8; 124.0; 123.8; 123.6; 122.6; 31.4; 27.9; 26.6; 22.4; 14.4. FTIR (KBr, *v*, cm^−1^): 3043 (C-H aromatic); 2952, 2929 (C-H aliphatic); 1695, 1664 (C=O imide); 1629 (-CH=N- imine). Anal. Calcd. for C_31_H_27_N_3_O_2_ (473.56 g/mol): C (78.62%) H (5.75%) N (8.87%); found: C (78.49%) H (5.52%) N (8.84%). 

3-(1-bromo-4-benzo)-*N*-hexyl-1,8-naphthalimide (AzNI-3)

Yellow solid. Yield = 63%. m = 0.29 g. ^1^H NMR (400 MHz, DMSO-*d*_6_, δ, ppm): 8.91 (s, 1H); 8.44 (dd, *J*_1_ = 12.4 Hz, *J*_2_ = 4.7 Hz, 3H); 8.31 (d, *J* = 2.0 Hz, 1H); 7.99 (d, *J* = 8.5 Hz, 2H); 7.89 (t, *J* = 7.8 Hz, 1H); 7.80 (d, *J* = 8.4 Hz, 2H); 4.13–4.00 (m, 2H); 1.72–1.55 (m, 2H); 1.40 - 1.23 (m, 6H); 0.87 (t, *J* = 6.9 Hz, 3H). ^13^C NMR (101 MHz, DMSO-*d*_6_, δ, ppm): 163.8; 163.6; 162.6; 150.0; 135.3; 134.6; 132.8; 132.5; 131.3; 130.4; 128.3; 126.3; 126.1; 125.4; 125.1; 123.7; 122.6; 31.4; 27.8; 26.6; 22.4; 14.4. FTIR (KBr, *v*, cm^−1^): 3063 (C-H aromatic); 2957, 2927 (C-H aliphatic); 1696, 1657 (C=O imide); 1630 (-CH=N- imine); 784 (-C-Br). Anal. Calcd. for C_25_H_23_BrN_2_O_2_ (463.37 g/mol): C (64.80%) H (5.00%) N (6.05%); found: C (64.40%) H (5.02%) N (5.95%).

3-(2,4-dichloro-4-benzo)-*N*-hexyl-1,8-naphthalimide (AzNI-4)

Yellow solid. Yield = 53%. m = 0.24 g. ^1^H NMR (400 MHz, DMSO-*d*_6_, δ, ppm): 9.02 (s, 1H); 8.48–8.38 (m, 2H); 8.32 (d, *J* = 14.4 Hz, 2H); 8.24 (d, *J* = 8.5 Hz, 1H); 7.86 (dd, *J*_1_ = 15.6 Hz, *J*_2_ = 7.6 Hz, 1H); 7.83–7.76 (m, 1H); 7.61 (dd, *J*_1_ = 8.5 Hz, *J*_2_ = 1.2 Hz, 1H); 4.12–4.06 (m, 2H); 1.70–1.52 (m, 2H); 1.39–1.18 (m, 6H); 0.87 (t, *J* = 6.5 Hz, 3H). ^13^C NMR (101 MHz, DMSO-*d*_6_, δ, ppm): 163.8; 163.6; 158.2; 149.8; 137.7; 136.6; 134.7; 132.8; 132.0; 130.6; 130.5; 130.2; 128.6; 128.3; 126.6; 125.5; 124.7; 123.8; 122.6; 31.3; 27.9; 26.6; 22.3; 14.2. FTIR (KBr, *v*, cm^−1^): 3061 (C-H aromatic); 2971, 2946 (C-H aliphatic); 1700, 1653 (C=O imide); 1628 (-CH=N- imine); 786 (-C-Cl). Anal. Calcd. for C_25_H_22_Cl_2_N_2_O_2_ (453.36 g/mol): C (66.23%) H (4.89%) N (6.18%); found: C (66.11%) H (4.95%) N (6.17%).

3-(4-nitro-4-benzo)-*N*-hexyl-1,8-naphthalimide (AzNI-5)

Yellow solid. Yield = 49%. m = 0.21g. ^1^H NMR (400 MHz, DMSO-*d*_6_, δ, ppm): 9.10 (s, 1H); 8.49–8.44 (m, 3H); 8.43–8.36 (m, 3H); 8.29 (d, *J* = 8.8 Hz, 2H); 7.90 (t, *J* = 7.8 Hz, 1H); 4.13–3.95 (m, 2H); 1.73–1.55 (m, 2H); 1.45–1.18 (m, 6H); 0.87 (t, *J* = 6.9 Hz, 3H). ^13^C NMR (101 MHz, DMSO-*d*_6_, δ, ppm): 163.7; 163.6; 162.0; 160.6; 149.6; 149.4; 142.0; 140.1; 134.7; 132.9; 130.7; 130.6; 130.0; 128.4; 126.7; 125.8; 125.1; 124.6; 123.8; 122.8; 31.4; 27.8; 26.6; 22.4; 14.3. FTIR (KBr, *v*, cm^−1^): 3073 (C-H aromatic); 2953, 2931 (C-H aliphatic); 1700, 1658 (C=O imide); 1628 (-CH=N- imine); 1522, (-NO_2_ stretch). Anal. Calcd. for C_25_H_23_N_3_O_4_ (429.47 g/mol): C (69.92%) H (5.40%) N (9.78%); found: C (69.69%) H (5.14%) N (9.81%).

3-(4-(1*H*-imidazol-1-yl)-4-benzo)-*N*-hexyl-1,8-naphthalimide (AzNI-6)

Yellow solid. Yield = 45%. m = 0.20 g. ^1^H NMR (400 MHz, DMSO-*d*_6_, δ, ppm): 8.96 (s, 1H); 8.45 (t, *J* = 7.7 Hz, 4H), 8.33 (s, 1H); 8.19 (d, *J* = 8.3 Hz, 2H); 7.90 (t, *J* = 8,0 Hz, 4H); 7.18 (s, 1H); 4.14–4.02 (m, 2H); 1.72–1.59 (m, 2H); 1.42–1.25 (m, 6H); 0.88 (t, *J* = 7.0 Hz, 3H). ^13^C NMR (101 MHz, DMSO-*d*_6_, δ, ppm): 164.2; 164.1; 162.7; 148.3; 142.2; 136.7; 134.9; 134.1; 131.9; 131.8; 131.1; 131.0; 127.4; 125.9; 123.1; 122.3; 121.2; 120.8; 118.3; 112.3; 31.4; 27.9; 26.4; 22.4; 14.2. FTIR (KBr, *v*, cm^−1^): 3056 (C-H aromatic); 2955, 2851 (C-H aliphatic); 1696, 1658 (C=O imide); 1618 (-CH=N- imine). Anal. Calcd. for C_28_H_26_N_4_O_2_ (450.53 g/mol): C (74.64%) H (5.82%) N (12.44%); found: C (74.30%) H (5.66%) N (12.04%).

### 2.3. Concentration Study

The concentration study was performed via dilution stock solution of the compounds in chloroform and methanol. First, on an analytical balance, the compounds were weighed and then dissolved in the test solvent to obtain a 100 μM solution. Thus, the stock solution was obtained. Then this solution was diluted in the appropriate proportions to obtain samples with the concentrations of the compound: 100, 80, 40, 20, 10, and 1 μM for the samples in chloroform and 100 and 10 μM for the samples in methanol. For AzNI-2, the preparation of a stock solution in methanol was not possible due to its poor solubility. Measurements were made 2 h after the samples were prepared.

### 2.4. Study of Spectroscopic Properties Related to Aggregation

The properties in the aggregated state were tested in a binary MeOH/H_2_O system, increasing the content of water (*fw*: 0, 10, 20, 30, 40, 50, 60, 70, 80, 90%, *v*/*v*). The appropriate masses of the test compounds were weighed then dissolved in dimethyl sulfoxide (DMSO) to obtain about 1.5 cm^3^ of a solution and a concentration 10 mM. Next, a kit of MeOH/H_2_O solvents (10 cm^3^ each) with different H_2_O content (*fw*: 0, 10, 20, 30, 40, 50, 60, 70, 80, 90%, *v*/*v*) was performed for each compound. Next, a 0.1 cm^3^ solution of the test compound was added to each system and vigorously mixed. After two hours, a measurement on a spectrofluorometer at an excitation wavelength of 340 and 430 nm was performed. After measuring, the vials were placed under a UV lamp (CAMAG, UV lamp 4, Muttenz, Switzerland)according to the increased H_2_O content in the system, and then excited at 366 nm, the photos were taken. The investigations were performed in pure MeOH (without DMSO content), stating no changes in the recorded spectra. Due to the better solubility in DMSO, the results are presented in the manuscript as described above.

### 2.5. Spectroscopic Studies of the Imine Bond Protonation by Trifluoroacetic Acid (TFA)

Spectroscopic studies of the protonation of the imine bond were performed in various solvents (chloroform, acetonitrile, and methanol) and various concentrations of trifluoroacetic acid (TFA). Test compounds were weighed and dissolved in DMSO to obtain approximately 2.5 cm^3^ of a 10 mM solution. TFA solution was obtained by weighing the acid and dissolving it in a suitable solvent (chloroform, acetonitrile, and methanol) to obtain about 15 cm^3^ of a 10 mM solution. Then, 8 cm^3^ of the solvent and 0.1 cm^3^ of the test compound solution were introduced into 10 cm^3^ volumetric flasks. Then, a solution of TFA in the tested solvent was added in the following amounts: 0, 0.01, 0.05, 0.1, 0.2, 0.3, 0.5, 1.5 cm^3^, respectively. Next, the flasks were made-up to the mark with the solvent. The mixture was mixed and left for 2 h. Afterward, measurements on a spectrofluorometer at excitation wavelengths of 340 and 430 nm were performed. After measuring, the vials were placed under a UV lamp according to the increased TFA content in the system, and then excited at 366 nm, the photos were taken.

### 2.6. Cell Lines and Culture Conditions

The human breast cancer cell line (MCF-7), the human colorectal carcinoma cell line (HCT 116), and the human glioblastoma cell line (U-87) were purchased from the ATCC (Manassa, VA, USA). The normal human dermal fibroblasts (NHDF) was obtained from PromoCell (Heidelberg, Germany). The cell lines were cultured in Dulbecco’s Modified Eagle Medium/Nutrient Mixture F-12 (DMEM/F-12) with 12% heat-inactivated fetal bovine serum (FBS) for tumor cells, or 15% non-inactivated FBS for NHDF, and a mixture of antibiotics: streptomycin and penicillin (1% *v*/*v*) (all reagents from Sigma-Aldrich, Saint Luis, MO, USA). All cell lines were grown under standard conditions at 37 °C and in a humidified atmosphere at 5% CO_2_. 

### 2.7. Cytotoxicity Studies

The cell lines were seeded at a density of 5000 cells per well (for MCF-7, HCT 116, U-87) and 4000 cells per well (for NHDF) on 96-well clear plates (Nunc) and incubated under standard conditions for one day. After 24 h, the complete DMEM was exchanged for solutions of AzNI compounds at concentrations ranging from 1 to 25 µM. After a 72 h incubation, the cytotoxicity assay—CellTiter 96^®^ AQ_ueous_ One Solution Cell Proliferation Assay–MTS (Promega, Medison, WI, USA)—was performed [28,35]. Briefly, the solutions of the tested AzNI compounds were removed, and 100 µL DMEM (without FBS and phenol red—PF) and 20 µL of MTS reagent were added and incubated for 1 h at 37 °C. Afterward, the optical densities of wells with controls (untreated cells) and tested compounds were measured at 490 nm using a multi-plate reader—Synergy 4 (BioTeK, Winooski, VT, USA). The obtained absorbance values are expressed as the percentage of the control; they were calculated as the inhibitory concentration (IC_50_) using GraphPad Prism 8 (GraphPad Software, San Diego, CA, USA). Each compound was tested in duplicate in one experiment with each experiment repeated three times.

### 2.8. The Cellular Staining

Before the cellular staining experiments, MCF-7 cells were seeded onto coverslips at a density of 50,000 cells per slide and incubated under standard conditions for 48 h. Afterward, the complete DMEM was exchanged for solutions of the tested 1,8-naphthalimide derivatives (25 μM). After 2 h incubation at 37 °C, the cells were washed three times with PBS and mounted with a DMEM without FBS and PF. The cellular staining results were immediately observed under 365 nm and 470 nm LED illumination (25% of power) using a Zeiss Axio Observer Z1 inverted fluorescence microscope (Oberkochen, Germany) equipped with an AxioCam MRm camera (Oberkochen, Germany). 

### 2.9. Co-Localization Studies

The co-localization experiments with some changes were performed according to the protocol described in [35]. Briefly, MCF-7 cells were seeded in the same manner as described in Section 2.8. Then, the medium was replaced with solutions of AzNI-3–6 (25 μM) and the breast cells were further incubated for 2 h. Afterward, the MCF-7 cells were rinsed with PBS. The medium (without FBS and PF) that contained MitoTracker^®^ Orange (Molecular Probes, Eugene, OR, USA) at a concentration of 100 nM or ER-Tracker™ Red BODIPY^®^ TR Glibenclamide (Molecular Probes, Eugene, OR, USA) at a concentration of 1 μM (both from Molecular Probes) were added and incubated for 30 min at 37 °C. After staining with mitochondria- or endoplasmic reticulum-specific dye, the breast cells were rinsed three times with PBS and mounted with a DMEM without FBS and PF. The subcellular localization results were immediately observed under 470 nm (for compounds), 550 nm (for mitochondria-dye), and 587 nm (for ER-dye) LED illumination (25% of power) using a Zeiss Axio Observer.Z1 inverted fluorescence microscope (Oberkochen, Germany). The obtained fluorescence images were processed using ImageJ software 1.41 (Wayne Rasband, National Institutes of Health, Bethesda, MD, USA). 

## 3. Result and Discussion 

### 3.1. Structural and Thermal Characterization 

**Scheme 1:**
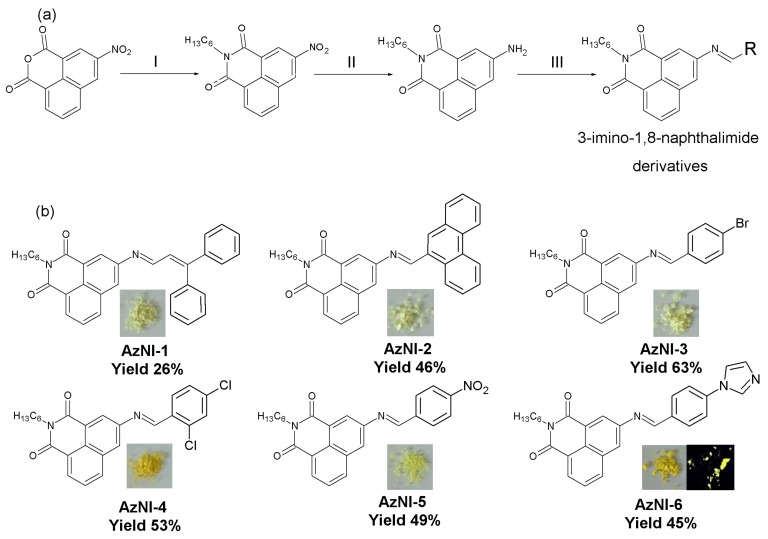
(**a**) The synthetic route of the 3-imino-1,8-naphthalimide derivatives: I—C_6_H_15_N,rfx,EtOH,6h, II—N_2_H_4_, Pd/C, rfx, EtOH, 24 h, III—MeOH, 24 h, aldehyde with (**b**) 1,8-naphthalimides derivatives chemical structure, photos of powder under daylight and for AzNI-6 under UV light (λ_ex_ = 366 nm).

The six new compounds were prepared by a simple one-step eco-friendly condensation reaction without any catalyst; they were obtained in a yellow solid state (powders, cf. Scheme 1). They were new compounds substituted in the naphthalene ring at the 3-C position via the π bond (-HC=N-). In this paper, the obtained materials are named in short as AzNIs and are presented in Scheme 1. They differ in substituents in the imine bond, which was linked with vinylene-1,1-diyldibenzene (AzNI-1), phenanthrene (AzNI-2), bromobenzene (AzNI-3), dichlorobenzene (AzNI-4), nitrobenzene (AzNI-5), and 1-phenyl-1*H*-pyrazole (AzNI-6) units. To fully prove the chemical structure of the synthesized *N*-hexyl-1,8-naphthalimide derivatives the ^1^H and ^13^C NMR, FTIR spectra and elemental analysis were used (the ^1^H NMR are available in the SI). COSY and HMQC spectra were registered for AzNI-1, as presented in Figure S2. The signal of the protons coming from the aliphatic chain were found in the range of 0.87–4.10 ppm. Moreover, the signal of the imine bond (-HC=N-) was observed at 8.20 ppm as a doublet, and at 7.16 ppm the signal of vinylene bond (-HC=C-) as a doublet was also seen. The absorption band in the FTIR spectra of -HC=N- linkage from 1626 to 1630 cm^−1^ was detected. The elemental analysis results confirmed the purity of the synthesized *N*-hexyl-1,8-naphthalimide derivatives. 

Thermal properties, which means thermal stability (investigated using thermogravimetric analysis) and temperature of phase transitions (with glass transition temperature, investigated using differential scanning calorimetry) are presented in Table 1.

**Figure 1:**
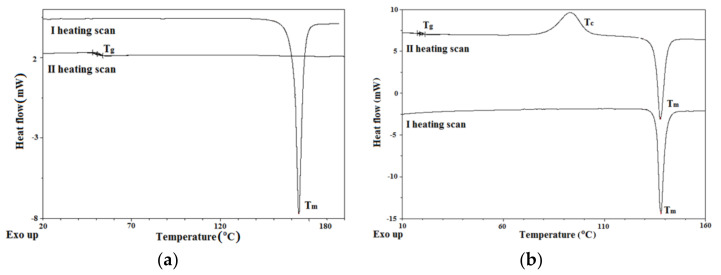
The differential scanning calorimetry (DSC) thermograms of (**a**) AzNI-2 and (**b**) AzNI-4.

The investigated AzNI were characterized by one-step of the decomposition with the maximum decomposition rate from 365 °C to 460 °C (cf. Figure S3 in the SI). They were thermally stable up to 280 °C, where 5% weight loss began at 288 °C for compound AzNI-6 with 1-phenyl-1*H*-pyrazole. The compounds with Br, Cl, and NO_2_ underwent thermal decomposition at similar temperatures as molecules with vinylene-1,1-diyldibenzene (AzNI-1). The introduction of phenanthrene (AzNI-2) significantly increased the thermal stability compared to the others substituents. The temperatures of the 5% weight loss were slightly lower than for the molecules with larger aromatic systems presented in our previous work [33]. From the differential scanning calorimetry thermograms, the endothermic peak of the melting temperature (T_m_) in the first heating scan was seen in the range of 120–164 °C (cf. Table 1 and Figure 1).

During the II heating scan (after cooling with 20 °C·min^−1^ rate), the glass transition temperature (T_g_) was revealed; the value was strongly dependent on the substituent attached to the imine bond. The synthesized crystalline molecules can be transformed into molecular glasses. Stable molecular glasses (without cold crystallization temperature and melting temperature during heating above T_g_) were obtained in the case of molecules with vinylene-1,1-diyldibenzene (AzNI-1), phenanthrene (AzNI-2), and 1-phenyl-1*H*-pyrazole (AzNI-6) substituent.

The thermal characterization was carried out in terms of the application of the tested compounds in prototype devices, registering a sufficiently high temperature of the beginning of thermal decomposition (T_5_) for this type of application.

### 3.2. Electrochemical Study

**Figure 2:**
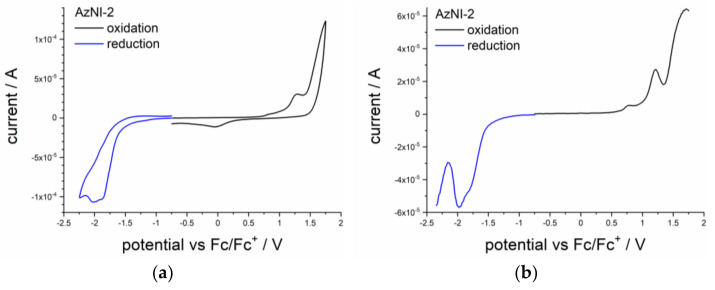
(**a**) Cyclic voltammetry (CV) and (**b**) differential pulse voltammetry (DPV) scans of AzNI-2 (scan rate 0.10 V/s, electrolyte 0.1 M Bu_4_NPF_6_ in dichloromethane).

In order to study the chemical activity of the AzNI compounds, we used cyclic voltammetry (CV) and differential pulse voltammetry (DPV). These two research methods helped to estimate the reduction and oxidation potentials and the energy band gap (E_g_) of the synthesized AzNI. The measurements were performed with a 0.1M Bu_4_NPF_6_ electrolyte in dichloromethane (c = 10^−3^ mol/dm^3^), with the platinum wire served as a working electrode and ferrocene couple (Fc/Fc^+^) as an internal standard. The cyclic voltammograms are presented in Figure 2. The outcome of ionization potentials (IP) and electron affinities (EA) (closely related to the HOMO and LUMO levels and later in the manuscript marked as E_HOMO_ and E_LUMO_), together with electrochemically derived energy band gap values, are presented in Table 2.

CV measurement allowed tracing the charge injection capabilities of the molecules. Two types of electrochemical experiments were performed for the AzNI series with two diverse polarization directions. The experiments were performed at the experimental scan rate of 0.10 V/s for CV and 0.05 V/s for DPV. It allowed trailing both electrochemical oxidation and reduction reactions induced by either removal or receiving an electron. The recorded data served to calculate the E_HOMO_ and E_LUMO_ by reading the onset potentials for the electrochemical reactions. Based on known E_HOMO_ of the ferrocene couple (equal to −5.1 eV [37]) the calculated energy levels are correlated to ionization potentials (E_HOMO_) and electron affinities (E_LUMO_) of the material by the simple mathematic relation. 

The structural arrangement of all investigated compounds comprise of the core 1,8-naphthalimide system, coupled with the adjacent bulky substituent with –C=N– linkage. Such architecture leads to the formation of similar structures to symmetric naphthalene diimide [38]. The electroactivity of the *N*-hexyl-1,8-naphthalimides derivatives was manifested by multi-step redox waves observed on both CV and DPV. They reflect the reduction of the naphthalimide part [39,40], prone to accept the electron, owing to its electron deficient nature [41] and oxidation of the donor unit.

The oxidation process is irreversible, where no peak on the return half-cycle of a CV is shown. This is indicative of chemical irreversibility of the system and might also be relevant to the rapid diffusion process occurring in the solution in the vicinity of the electrode. Oxidative ability depends on the chemical structure of the donor fragment. The lowest oxidation potential value was recorded for AzNI-4 (dichlorobenzene moiety) and AzNI-2 (phenanthrene unit), which present their willingness to lose the electron. On the other hand, the highest oxidation potential of AzNI-1 (vinylene-1,1-diyldibenzene substituent) reflects the system’s reluctance to lose the electron. The reduction peaks were registered in the range of E_red_ = −1.94 V to E_red_ = −1.57 V (cf. Table 2) for the acceptor part; moreover, the half-peak redox potential of the naphthalimide was found at −1.71 V vs. Fc/Fc^+^ and was described in publication [42]. The shift of the reduction peak potential results from the electron impact induced by donor fragments of the molecules, which can either lower the observed potential (as for AzNI-1, AzNI-2, AzNI-3, and AzNI-4) or raise it (as for AzNI-5 and AzNI-6). The strongest effect was observed for the AzNI-4 endowed doubly substituted benzyl ring. For some of the compounds, the reduction reaction was quasi-reversible, namely for AzNI-3 and AzNI-5. Such behavior depends on competition between the mass transport events and kinetics of the heterogeneous electron transfer observed during CV experiments.

Close inspection of the electrochemically derived energy gap values revealed the lowest E_g_^CV^ for AzNI-6 (1-phenyl-1*H*-pyrazole substituent), mainly induced by high energy of the HOMO level. The compounds were ranked in the following order of increasing value of E_g_: AzNI-6 < AzNI-3 < AzNI-5 < AzNI-2 < AzNI-4 < AzNI-1. In the studied group, a more profound effect was observed on the E_LUMO_ (and, hence, EA) value as it changes within the range of 0.55 V, while the E_HOMO_ spans within the range of 0.34 V. 

### 3.3. Luminescence Investigations

**Figure 3:**
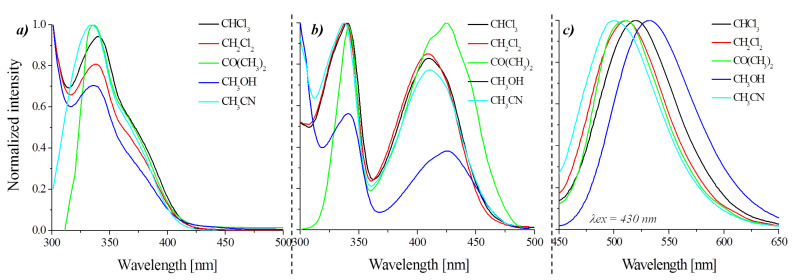
The (**a**) absorption, (**b**) excitation, and (**c**) emission spectra of AzNI-2 in various solvents.

The absorption, excitation, and emission measurements were performed in non-polar, polar, and polar protic solvents, such as chloroform (CHCl_3_, ε = 4.89), dichloromethane (CH_2_Cl_2_, ε = 8.93), acetone (CO(CH_3_)_2_, ε = 20.56), methanol (CH_3_OH, ε = 32.66), acetonitrile (CH_3_CN, ε = 35.94). The absorption and emission measurements were also performed in a solid state as a thin film and blend with PVK:PBD (50:50 wt.%) containing the 2 or 15 wt.% of AzNIs. The poly(*N*-vinylcarbazole) (PVK) and (2-tert-butylphenyl-5-biphenyl-1,3,4-oxadiazole) (PBD) mixed together (50:50 wt.%) form a two-component matrix. The electroluminescence (EL) ability of the investigated *N*-hexyl-1,8-naphthalimides derivatives was tested with the same composition as the blend. The registered absorption, excitation, and emission spectra are presented in Figure 3 and Figures S5–S10, while the spectroscopic data are presented in Table 3.

The analysis of the excitation spectra of the tested compounds in solutions revealed two wavelength ranges, i.e., about 320–350 nm and 380–475 nm, regardless of the polarity of the solvent (cf. Figure 3 and Figure S5). AzNI were absorbed with λ_max_ in the range 330–440 nm (cf. Table 3) with λ_max_ about 330 nm belonging to π → π* naphthalimide [33,36]. The PL emission of AzNI in the chloroform solution ranged from 514 to 524 nm, while in the remaining non-protic solvents, a shift of the wavelength to a higher energy, covering the range of 492–511 nm, was noted (cf. Table 4, Figure 3). In turn, the maximum emission for each compound in methanol was redshifted with respect to other solvents in the range of 525–548 nm, which is due to the intermolecular hydrogen bonding with protic solvents [28,34].

The compounds generally show a low emission intensity (cf. Table 4, Φ at about 0.6–3.2%), mainly due to the photoinduced electron transfer (PET) process [5,12,43]. Nevertheless, in order to compare the influence of the compound structure (donor type) on the optical properties in the solution, 3D photoluminescence spectra made in three solvents (CHCl_3_, CH_3_CN, CH_3_OH) were compiled, with the same concentration of the compound, and performing the measurement under the same conditions. The superimposed spectra are shown in Figure S10. The compounds AzNI-1 and AzNI-4 showed the weakest emission intensity in the tested solutions. For AzNI-2,5 and 6, intense emission was visible; however, in acetonitrile and methanol, its intensity decreases. However, for AzNI-3, the polarity of the solvent is also important, because in acetonitrile and methanol, a slight increase in photoluminescence was noticeable. On this basis, it can be concluded that the observed solvatochromism is associated with slight changes in the emission maximum, but, undoubtedly, the type of solvent affects the intensity of emission, depending on the donor-acceptor system. The discussion continues in Section 3.3.2, where the influence of the environment on the protonation of an imine bond in the solutions was investigated.

**Figure 4:**
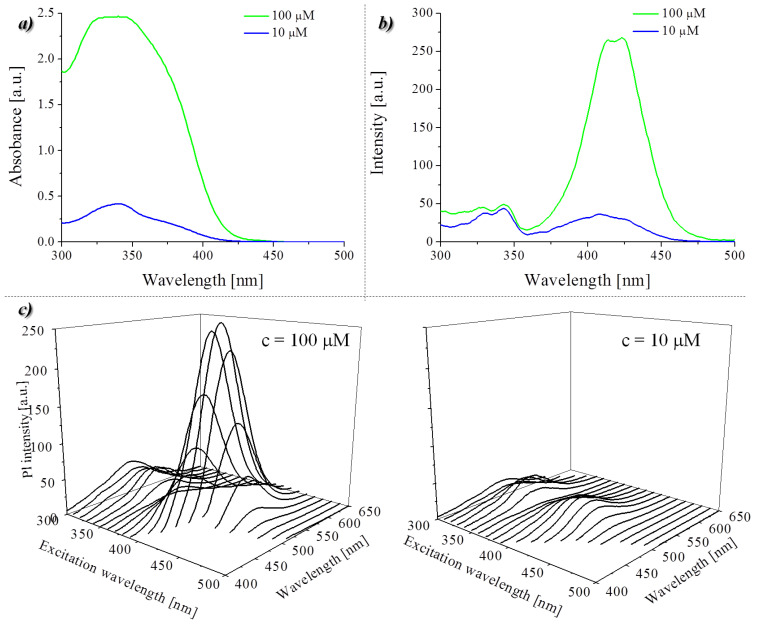
The (**a**) absorption, (**b**) excitation, and (**c**) 3D fluorescence spectra of AzNI-2 in chloroform solution by different concentration (c = 100, 80, 40, 20, 10, 1 μM).

The influence of the concentration of AzNIs in chloroform and methanol solutions was also investigated. The concentration range in chloroform was 100, 80, 40, 20, 10, and 1 μM, and in methanol, 100 and 10 μM. The results of these tests are presented in Figure 4 and in the SI (cf. Figures S16 and S17).

Analyzing Figure 4c, it is observed that, at a higher concentration of compounds (c = 100 μM), the intensity of the band increases in the excitation range from 400 to 480 nm. This band was not observed in the absorbance spectra. However, at a lower concentration (c = 10 μM), the intensity of this band significantly reduced. It is also illustrated by the excitation spectra shown in Figure 4b. On the recorded absorption spectra (Figure 4a), it was not possible to observe changes that are visible in the excitation spectrum as well as in the 3D photoluminescence spectra. In Figure S17, it can be seen that the intensity of the band at the excitation of λ_ex_ = 430 nm depends more on the concentration of the compound than at the excitation of 340 nm. Therefore, it should be assumed that the observed properties do not concern the compound properties, but only strong interactions between them. Thus, the increase of the emission intensity in the range of 400 to 480 nm, with the increase of the compound concentration in the system, may be due to interactions between the molecules, e.g., by the formation of excimers [44,45]. 

The absorption and emission spectra were registered in films (cf. Figure S6); blends with PVK:PBD (poly(*N*-vinylcarbazole):2-tert-butylphenyl-5-biphenyl-1,3,4-oxadiazole) matrix exhibited weak PL or were non-emissive in the solid state. The blends with PVK:PBD showed typical absorption at 310 and 344 nm as shoulders belonging to the PVK:PBD matrix (cf. Figure S7). The two emission bands, about 390 nm of the matrix and in the lower energy, were seen in blends (cf. Figure S7), as reported earlier in [33,36]. In the matrix (host) and the guest (compounds), the energy transfer from the host to the guest in the ground state can take place (by Dexter (exchange) or Förster (dipole–dipole interactions) mechanism) [46]. Full effective energy transfer can be seen when the emission intensity of the host decreases and the guest increases (the host presence is needed) and the host emission overlaps with the absorption spectrum of the guest [36,46]. For the *N*-hexyl-1,8-naphthalimides, the partial overlap, and two PL bands were seen, as presented in Figure S7 in the SI. The presence of the two PL bands indicated no complete energy transfer from the host (PVK:PBD matrix) to the guest (AzNIs) via Förster energy transfer [33,36]. 

#### 3.3.1. Photoluminescence in Aggregated State

Compounds with a tendency to aggregation-induced emission (AIE) are sought after, mainly due to the possibility of their wide application in organic electronics, i.e., OLEDs or cellular imaging [2]. Sometimes an insignificant change of properties allows AIE-gens to be obtained [47,48,49,50]. Therefore, as part of the work, we present research on the properties of compounds in the aggregated state. The tests were carried out in a binary MeOH/H_2_O system, with a different content of water fractions and the same concentration of the tested compound.

**Figure 5:**
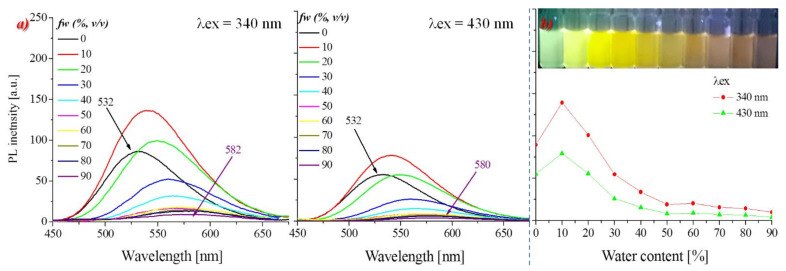
PL properties of AzNI-2 in a binary mixture of MeOH/H_2_O: (**a**) with an increasing water content (*fw*) at excitation of 340 and 430 nm; (**b**) λ_em_ intensity vs. the water content (*fw*) in the solvents mixture. Photographs were taken under λ = 366 nm UV irradiation from a hand-held UV lamp.

The obtained results are presented in Figure 5 and Figure S11. All compounds initially showed an increase in emissions; however, with a higher water content in the system, it was significantly reduced. Moreover, the red shift of the emission is also visible, which is well reflected in the photos taken under the UV lamp. Therefore, all compounds showed a tendency to aggregation caused quenching (ACQ) with increased water content in the system. The results shown in this work are slightly different from those described in our earlier work. In the case of azomethine with a hydroxyl group, the significance of the excited state intramolecular proton transfer (ESIPT) with protic solvents, with a tendency towards ACQ, was described [28], as well as the importance of the hydrolysis of this system related to the presence of an active site complexation [25,26,28]. In contrast, analogs containing a β-ketoenamine linkage showed a tendency towards AIE [35].

#### 3.3.2. Photoinduced Electron Transport Inhibition Process

**Figure 6:**
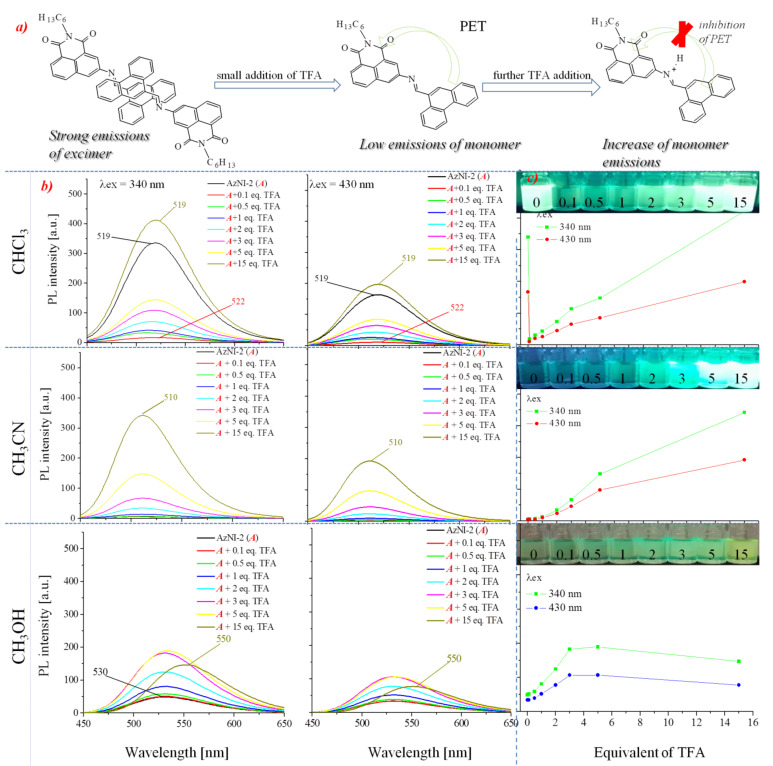
Effect of the TFA on photoluminescence (PL) properties of AzNI-2 in chloroform, acetonitrile, and methanol: (**a**) the proposed mechanism; (**b**) superimposed emission spectra at excitation 340 or 430 nm; (**c**) λ_em_ intensity versus equivalent of TFA. Photographs were taken under λ = 366 nm UV irradiation from a hand-held UV lamp.

In this part of the work, the inhibition of the PET process by the protonation of the imine bond using trifluoroacetic acid (TFA) in various solvents was investigated. The tests were carried out for all compounds at a concentration of 10^−5^ mol/dm^3^ and with the addition of TFA in molar ratios ranging from 0.1 to 15 equivalents, as well as in various solvents, such as chloroform (CHCl_3_), acetonitrile (CH_3_CN), and methanol (CH_3_OH). Moreover, the amine (substrate for the target compounds) was tested in the same solvents and TFA concentration. This substrate was tested due to the possible hydrolysis of imines [25,26,28] as well as the use of an amine in cellular imaging reported in our earlier studies [28]. The results for amine are shown in Figure S9. In these studies, there was no significant influence of TFA in any of the solvents on the photoluminescent properties of the amine. Whereas, the results for the target compounds are shown in Figure 6 and Figures S12–S15. It was observed that, by using a small amount of TFA in chloroform, the photoluminescence decreased, which may be indicated by the formation of the excimer in the pure solvent [34,51]. Accordingly, the formation of excimers mainly affects AzNI-2,5 and 6. Moreover, this phenomenon was not observed in acetonitrile, which explains the low photoluminescence of all compounds (Figure S11). In the next step, the addition of TFA caused an increase in emissions, which was caused by the protonation of the imine bond and inhibition of the PET process. Changes in the intensity of photoluminescence, due to the inhibition of PET processes, were most visible in non-protic solvents, such as, chloroform and acetonitrile (Figure 5, Figures S12 and S13).

The results are slightly different in the protic solvent (MeOH), where for the compounds AzNI-2,3,4,6 there is an increase in photoluminescence in contrast to AzNI-1 and 5. Moreover, these compounds show photoluminescence in methanol (Figure S10) and their photoluminescence increases with the addition of TFA. This may indicate easy protonation of the imine bond (even by a protic solvent) for donor acceptor systems containing the appropriate substituents. Moreover, for the compound AzNI-6 at λ_ex_ = 340 nm, double emission was observed with a maximum at 436 and 540 nm, which is visible in more polar solvents (Figure S15). Nevertheless, the addition of TFA causes the band intensity to increase at 540 nm, which clearly shows the change of the color of the solution from blue to green. Thus, the results of these studies show that the PET inhibition in the 3-imine-1,8-naphthalimide derivative largely depend on the donor used. In our research, the vinylene-1,1-diyldibenzene and nitrobenzene substituents adversely affected the ability to protonate the imine bond in methanol and increase of emissions in this system was no observed. In the remaining cases, the inhibition of the PET process occurred in the protic solvent itself, as well as with a further increase in TFA in the system. Moreover, it should be noted that studies in the MeOH/H_2_O binary systems (Figure S11) indicate that, initially, the addition of water caused an increase in emissions. Such an influence of the environment on the emission properties due to the visible increase in emission we have for the same compounds, i.e., AzNI-2,3,4,6. On the other hand, for AzNI-1 and AzNI-2 compounds, a slight increase in emission took place with a higher water content in the binary system.

#### 3.3.3. Luminescence Induced by External Voltage

**Figure 7:**
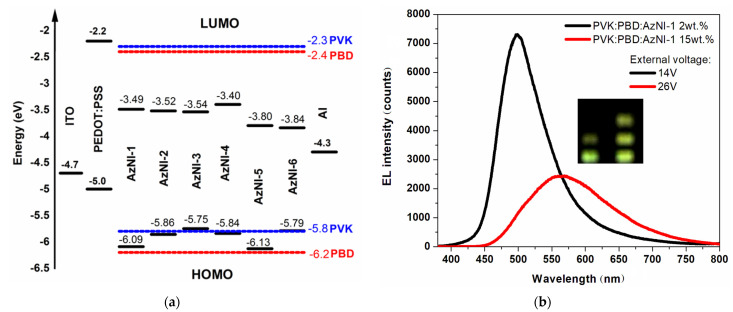

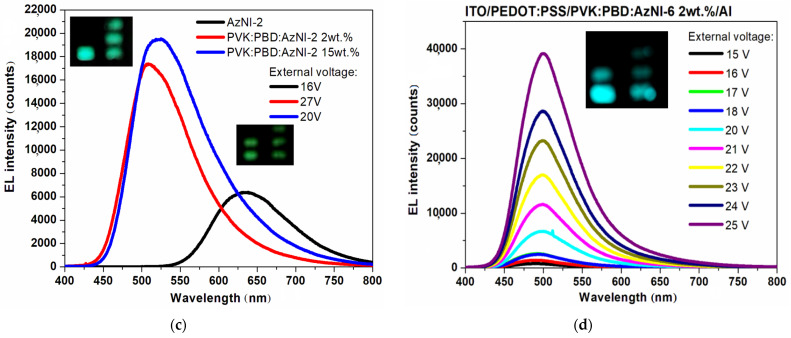
(**a**) The energy level diagram and EL spectra recorded from the working devices with (**b**) AzNI-1, (**c**) AzNI-2, and (**d**) AzNI-6 with their photo under external voltage.

The capacity of the new 3-imino-1,8-naphthalimide derivatives for the emission of light induced by voltage were examined in diodes with structure: GLASS/ITO/PEDOT:PSS/AzNI/Al and with guest-host configuration: GLASS/ITO/PEDOT:PSS/PVK:PBD:AzNI (2wt.% or 15wt.%)/Al. The PVK:PBD matrix was used as the host material to create the emitter (guest)–matrix (host) structures. The two-component matrix (PVK:PBD) exhibited good hole and electron mobility [46]. For that configuration, the singlet excitons (creating an electron-hole pair) formed in the PVK:PBD matrix can be transferred to the guest molecules, what was explained earlier in the text. The use of the matrix allowed increasing the probability of the occurrence, the excitons recombination and, thus, recording the electroluminescence spectrum, which was confirmed in this study. Only for one device (cf. Table 4), with the active layer containing a neat AzNI with the phenanthrene unit (AzNI-2), the maximum of the electroluminescence band (λ_EL_) was seen in the red light range (λ_EL_ = 636 nm, cf. Figure 7). For the other two devices with various content of AzNI-2 the λ_EL_ in the blue and green visible spectral region was seen. Devices with the 2wt.% content of the AzNI derivatives emitted blue light, and with the 15wt.% content of the guest emitted green light except for diode with AzNI-3 (bromobenzene substituent) and AzNI-5 (nitrobenzene substituent). 

The highest EL intensity was registered for devices with AzNI-3, and the lowest for devices with AzNI-1 (vinylene-1,1-diyldibenzene) (cf. Table 4). At this stage of research, only the electroluminescence (EL) spectra were registered.

The host–guest energy transfer (via the Förster mechanism) can occur in the presented devices. However, the energy transfer between PVK:PBD matrix (the host) and AzNI (the guest) is not efficient. Two bands in the emission spectra was seen, from the matrix and from the molecules. In this case, it can be assumed that the mechanism of EL is mixed, charge trapping mechanism (because the HOMO and LUMO energy levels of the AzNI molecules are between the HOMO and LUMO energy levels of the hosts) with the partially effective Förster mechanism [33,36].

In our previous research [33,35,36], the electroluminescence study was also performed. For unsymmetrical imino-imides [33,35], the maximum of the electroluminescence band (λ_EL_) was seen from the blue to orange light range for the PVK:PBD:imino-imides active layer configuration. The λ_EL_ in the red light range was seen for devices with active layer content neat imino-imides. More perspective compounds in OLED applications are bis-imino-imides as previously described [36], where the λ_EL_ for devices with neat bis-imino-imides were in the red spectral region (approximately λ_EL_ = 670 nm). Unsymmetrical imino-imides acting as an active layer in OLEDs exhibited λ_EL_ approximately 640 nm [35]. From this research, the perspective compounds for further investigations and modification are compounds with phenanthrene (AzNI-2) and bromobenzene substituent (AzNI-3) with the highest EL intensity. 

### 3.4. Biological Studies

The favorable optical properties of tested *N*-hexyl-1,8-naphthalimide derivatives containing an imine bond prompted us to explore their applicability in cell imaging. In addition, our previous works demonstrated interesting cellular behavior for a series of 1,8-naphatalimides containing an imine or β-ketoenamine bond and different substituents in the imide part or the naphthalene ring, whose ability to hydrolysis or aggregation was crucial for imaging [28,35]. It is worth mentioning that fluorescent compounds that can be potential cellular dyes must be characterized by several important parameters, such as the large Stokes shift above 80 nm, excitation above 340 nm, high fluorescence quantum yield, low photobleaching, interaction with proteins, as well as suitable lipophilicity and slightly amphiphilic character. These elements can be crucial to achieving a low signal-to-noise ratio and high-quality images. Nevertheless, the proper behavior and low toxicity of the compounds in the cellular environment is also valuable. With this in mind, we first performed cytotoxicity studies of all synthesized compounds on a panel of cancer cell lines representing different origins: breast (MCF-7), colon (HCT 116), and brain (U-87). The cancer cells were incubated with a wide range of concentrations of the 1,8-naphtalimides that were tested for 72 h. The influence of the compounds on cell viability was determined using an MTS assay based on tetrazolium salt. The cytotoxicity results expressed as IC_50_ values, defined as the concentration of the compound required for 50% inhibition of cell viability, are presented in Table 5. Additionally, for diagnostic applications, the fluorescent compounds considered as cellular dyes should have a high safety profile on normal cells. For this purpose, we examined all of the derivatives for cytotoxicity against the normal human dermal fibroblast (NHDF) cell line. In general, the tested 1,8-naphtalimides did not exhibited cytotoxicity on cancer and normal human cells. In addition, the tested compounds at a concentration of 25 µM showed no effect on cell viability during the 72 h duration of the experiment, allowing them to be used for long-term staining in a variety of cellular systems.

#### Cell Imaging

**Figure 8:**
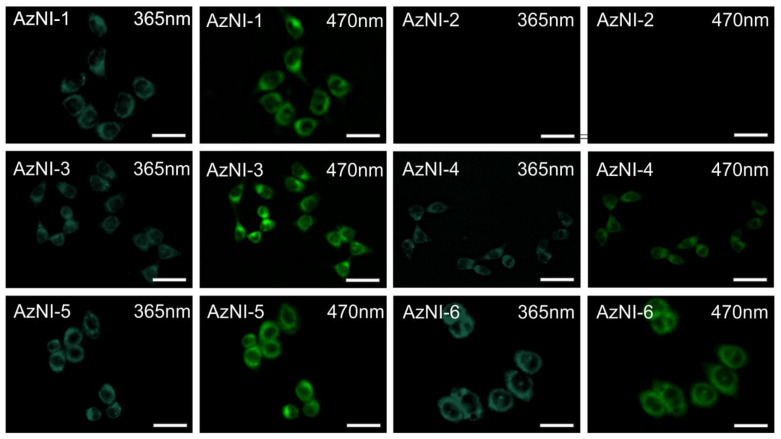
The fluorescence images of MCF-7 cells that were stained with the tested 1,8-napthalimide compounds (25 µM) for 2 h. The images acquired under 365 and 470 nm LED illumination. Scale bars indicate 25 µm.

To evaluate the behavior of the tested *N*-hexyl-1,8-naphthalimides in a cellular environment, the authors performed a series of cellular imaging experiments on breast cancer cells—MCF-7. Our preliminary data revealed that the tested derivatives reach their maximum fluorescence level in cells after 1–2 h incubation. This time is relatively quick and comparable to a variety of commercially available fluorescent probes. Interestingly, the spectroscopic profile and behavior in the aquatic environment of tested compounds allow excitation with two filters: DAPI (excitation at 365 nm wavelength) and GFP (excitation at 470 nm wavelength) in microscopic experiments. The advantages of excitation in green light in molecular imaging are a reduced signal-to-noise ratio and a very low background level from autofluorescence emitted by cells. Generally, in microscopic observations, greater autofluorescence is observed for shorter excitation wavelengths, especially in the UV region. Fluorescence images of the tested AzNIs under LED illumination at two wavelengths (365 and 470 nm) are presented in Figure 8.

**Figure 9:**
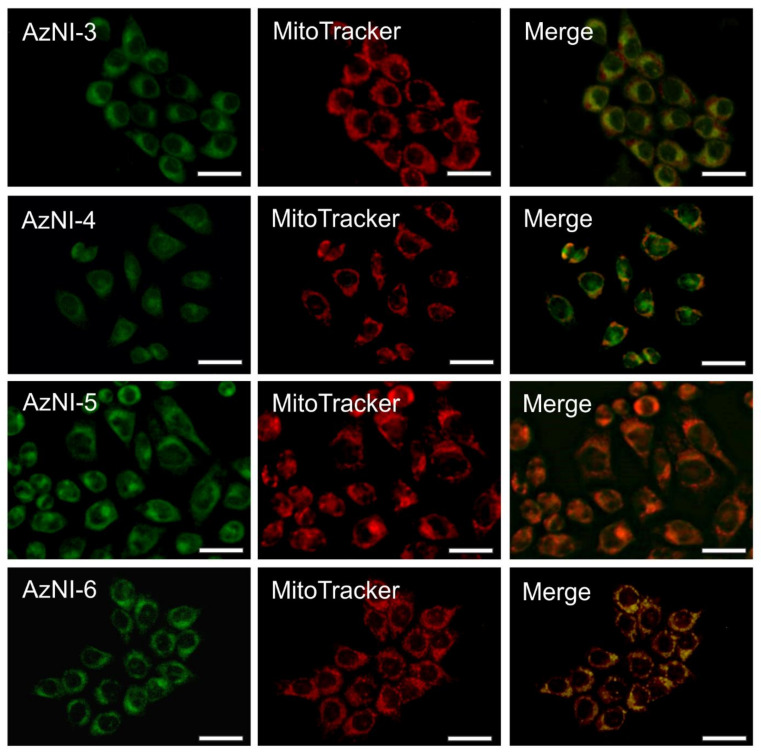
Co-localization fluorescence images of AzNI-3–6 at a concentration of 25 µM and MitoTracker Orange dye in MCF-7 cells. The images acquired under 470 nm (for AzNIs) and 550 nm (for mitochondria-dye) LED illumination. Scale bars indicate 25 µm.

In general, we observed a strong fluorescence signal in breast cells after incubation with most of the 1,8-naphthalimides that were tested. In the DAPI excitation filter, these compounds emitted blue–green fluorescence, whereas we recorded a green signal in the GFP filter. For the AzNI-2 derivative with the phenanthrene substituent, we did not register any fluorescence signal after excitation in the two filter. The lack of observed fluorescence signal in cells may have several causes. One of them may be related to the lack of penetration of the AzNI-2 across the cell membrane; thus, preventing the compound from entering the cell. The other may be due to an excessive ability of the compound to aggregate in compartments and the cellular environment. As we have shown in spectroscopic measurements (Figure 5), all compounds may exhibit a tendency to aggregation caused quenching (ACQ) in a system with increased water content. Nevertheless, the interaction of the phenanthrene substituent attached to the naphthalene ring at the 3-C position via the imine bond, with metal ions, proteins, or other structures that may cause fluorescence quenching, may also be important [52]. Moreover, our cellular staining experiments seem to clearly indicate the ability of 1,8-naphthalimides to accumulate in membrane compartments in the cell. Similar results were observed in our previous work for 3-imino-(2-phenol)-1,8-naphthalimide, where we registered accumulation in mitochondria, endoplasmic reticulum, and lysosomes [35]. Therefore, in further cellular studies, we decided to perform co-localization experiments for the four derivatives with organelle-specific dyes, such as MitoTracker Orange or ER-Tracker Red. As presented in Figure 9, the tested compounds showed similar behavior to accumulation in the cellular organelles. All of the tested 1,8-napthalimides displayed a tendency to bind to the negatively charged mitochondrial membrane. These results are consistent with numerous reports of the high affinity of 1,8-naphthalimide derivatives for staining the mitochondria of the cell [28,35,53]. Additionally, the AzNI-3 containing a bromophenol group seemed to have an affinity to the endoplasmatic reticulum (Figure S18 in the SI). This localization result can be explained by the more lipophilic nature of the bromophenol derivative, which is consistent with literature data [28,54]. The results may also explain the close contact between the endoplasmic reticulum and mitochondrial membrane protein, which formed a well-organized structural-network called mitochondria-associated membranes (MAM) [55]. On the other hand, in the case of AzNI-4 with a dichlorophenol moiety, we registered a signal in the cells’ central region, which did not overlap with the signal from the mitochondria-specific dye. It appears that this signal may come from the cell nucleus. Similar co-localization studies may support this hypothesis for terpyridine containing dichlorophenol substituent or 1,8-naphalimide derivatives, which nucleus-targeted imaging [56,57]. In summary, the tested 3-imino-1,8-naphatalimides may be interesting imaging tools and fluorescent materials for diagnostic applications and molecular bioimaging.

## 4. Conclusions 

A series of six novel 1,8-naphthalimide imine derivatives containing various donor substituents in the 3-C position of the naphthalene ring were synthesized and characterized. The effect of the substituent structure on thermal, electrochemical, luminescence, and biological properties was demonstrated. The phenanthrene structure increases the T_m_ and thermal stability. The electrochemical studies showed that introduction of the nitrobenzene (AzNI-5) and 1-phenyl-1*H*-pyrazole (AzNI-6) unit lowered electron affinity compared to the others substituents. Additionally, the presence of 1-phenyl-1*H*-pyrazole (AzNI-6) and bromobenzene (AzNI-3) lowered E_g_ electrochemically estimated to 1.95 and 2.21 eV, respectively. Generally, AzNIs exhibited very low PL quantum yield due to the PET process. The most intense emission induced by radiation was observed in chloroform solution with similar λ_em_, about 520 nm. *N*-hexyl-1,8-naphthalimes substituted with phenanthrene (AzNI-2), bromobenzene (AzNI-3), and 1-phenyl-1*H*-pyrazole (AzNI-6) were photoluminescent in the solid state as thin film. Moreover, for AzNI-2 and AzNI-6, the highest PL Φ (above 3.2%) was measured. Spectroscopic studies showed that all compounds had a tendency to aggregation-caused quenching of photoluminescence in the MeOH/H_2_O system, as well as increase the emission due to the inhibition of the PET process, which primarily depended on the substituent structure and the solvent type, especially the polar and protic environment. Considering the PL quantum yield of bands corresponding to matrix and AzNI emission, in the case of AzNI-3 (2 wt.% in blend) and AzNI-6 (15 wt.% in blend), the Förster energy transfer seems to be the most efficient. Only the molecule with phenanthrene (AzNI-2) applied as an active layer in the diode emitted light. On the other hand, all devices with AzNI dispersed molecularly in the matrix were emissive; however, the maximal EL intensity was obtained under very high voltage. Thus, they cannot be considering in the PVK:PBD matrix as electroluminescent materials. The biological study revealed that *N*-hexyl-1,8-naphthalimides derivatives showed no biological activity against normal human dermal fibroblast (NHDF) and cancer (MCF-7, HCT 116, U-87) cells. However, most of the compounds were successfully used in cancer (MCF-7) cellular imaging. At an excitation of 365 nm (DAPI filter), cells were stained on blue–green, with excitation at 470 nm (GFP Filter) on green. A tendency mainly towards staining of the mitochondria was demonstrated in colocalization studies. The cell imaging results obtained was different from those obtained with the amine, where staining was possible only at 365 nm excitation (DAPI filter), getting a green color. Thus, PET inhibition is responsible for the obtained results of cellular imaging. It is emphasized by the specificity of staining, other than by using the substrate (amine), as well as ACQ properties of the test compounds. The authors consider the studied compounds as “prospective” in cell imaging. In the future, we plan to extend biological research and modify the chemical structure of the presented compounds in terms of cell imaging.

## Figures and Tables

**Table 1:** Thermal properties—thermal stability and temperatures of phase transitions (with glass transition temperature) of the investigated molecules.

Molecules	TGA			DSC			
				Scan I	Scan II		
	T_5_^a^	T_10_^a^	T_max_^b^	T_m_^c^	T_g_^e^	T_c_^d^	T_m_^c^
	(°C)	(°C)	(°C)	(°C)	(°C)	(°C)	(°C)
AzNI-1	337	354	388	143	35	nd	nd
AzNI-2	365	387	460	164	51	nd	nd
AzNI-3	291	312	365	120	18	78	120
AzNI-4	297	322	376	138	18	93	138
AzNI-5	309	326	374	153	34	72	152
AzNI-6	288	327	397	154	53	nd	nd

^a^ T_5_ and T_10_ means temperature based on 5% and 10% weight loss from TGA curves. ^b^ T_max_ means temperature of the maximum decomposition rate from the first derivative of the TGA curves. ^c^ T_m_ means melting temperature ^d^ T_c_ means cold crystallization temperature. ^e^ T_g_ means glass transition temperature. Scan I and Scan II mean the first and second (after cooling) heating scan, respectively. nd means not detected.

**Table 2:** Electrochemical investigation results of the investigated *N*-hexyl-1,8-naphthalimides derivatives.

Molecule	Method	E_red_^a^	E_red(onset)_^a^	E_ox_^a^	E_ox(onset)_^a^	E_LUMO_	E_HOMO_	E_g_
		(V)	(V)	(V)	(V)	(eV)	(eV)	(eV)
AzNI-1	CV ^b^	−1.91	−1.61	1.33	0.99	−3.49	−6.09	2.60
	DPV ^c^	−1.88	−1.55	1.12	0.96	−3.55	−6.06	2.52
AzNI-2	CV	−1.87	−1.58	0.83	0.76	−3.52	−5.86	2.34
	DPV	−1.80	−1.52	0.77	0.68	−3.58	−5.78	2.20
AzNI-3	CV	−1.86	−1.54	0.88	0.65	−3.56	−5.75	2.21
	DPV	−1.85	−1.53	0.87	0.55	−3.57	−5.65	2.23
AzNI-4	CV	−1.94	−1.70	0.82	0.74	−3.45	−5.84	2.44
	DPV	−2.03	−1.67	0.72	0.52	−3.46	−5.62	2.19
AzNI-5	CV	−1.57	−1.27	broad wave	1.03	−3.83	−6.08	2.33
	DPV	−1.36	−1.14	0.77	0.55	−3.96	−5.62	1.69
AzNI-6	CV	−1.58	−1.36	0.92	0.69	−3.74	−5.79	1.95
	DPV	−1.44	−1.21	0.86	0.65	−3.89	−5.75	1.86

**E_HOMO_** = –5,1–E_ox(onset)_·|e|, **E_LUMO_** = –5,1–E_red(onset)_·|e|, E_g_ = E_ox(onset)_ –E_red(onset)_. Measurements in CH_2_Cl_2_ with c_sample_ = 10^−3^ mol/dm^3^ and c_electrolyte_ = 0.1 mol/dm^3^ (Bu_4_NPF_6_), Pt as the working electrode. ^a^ Potentials were taken from the first oxidation/reduction processes. ^b^ V = 0.10 V/s. ^c^ V = 0.05 V/s.

**Table 3:** The photoluminescence data of the investigated AzNI compounds.

Molecules	Medium	UV-Vis λ_max_ (nm) (ε∙10^4^) ^a^	λ_ex_ (nm)	λ_em_ (nm)	Φ (%)	τ (ns)
AzNI-1	CHCl_3_	340 (4.02), 388 ^sh^	337, 410	519	0.6	12.03
	CH_2_Cl_2_	338 (4.59), 386 ^sh^	334, 412	500, 524 ^sh^	-	-
	CO(CH_3_)_2_	338 (4.15), 380 ^sh^	326, 380	495, 515 ^sh^	-	-
	CH_3_OH	338 (4.46), 383 ^sh^	341, 430	541	-	-
	CH_3_CN	336 (4.20), 381 ^sh^	336, 422	500, 513	-	-
	Blend PVK:PBD ^b^	310 ^sh^, 344 ^sh^	^-^	378, 476	3.6, 2.1	-
	Blend PVK:PBD ^c^	310 ^sh^, 344 ^sh^	-	380, 486	2.9, 1.4	-
AzNI-2	CHCl_3_	346 (3.87), 385 ^sh^	329 ^sh^, 341, 410, 427 ^sh^	519	3.5	14.0
	CH_2_Cl_2_	345 (3.95), 380 ^sh^	329 ^sh^, 340, 408, 427 ^sh^	510	-	-
	CO(CH_3_)_2_	342 (3.93), 380 ^sh^	341, 410 ^sh^, 425	507	-	-
	CH_3_OH	342 (3.48), 378 ^sh^	326 sh, 340, 407 ^sh^, 425	532	-	-
	CH_3_CN	340 (3.67), 378 ^sh^	322 ^sh^, 340, 409, 423 ^sh^	495	-	-
	Film	342	-	525	3.5	-
	Blend PVK:PBD ^b^	310 ^sh^, 344 ^sh^	-	379, 477	4.6, 3.2	-
	Blend PVK:PBD ^c^	310 ^sh^, 344 ^sh^	-	379, 493	5.9, 2.5	-
AzNI-3	CHCl_3_	328 (2.86), 346 (0.82)	325 ^sh^, 342, 408, 425 ^sh^	520	1.2	12.0
	CH_2_Cl_2_	342 ^sh^, 382 ^sh^, 428 (0.18)	326 ^sh^, 340, 409, 423 ^sh^	510	-	-
	CO(CH_3_)_2_	336 (2.23), 380 ^sh^, 434 (0.13)	341, 410 ^sh^, 425	510	-	-
	CH_3_OH	347 ^sh^, 379 ^sh^, 440 (0.23)	342, 408 ^sh^, 426	540	-	-
	CH_3_CN	338 ^sh^, 378 ^sh^, 426 (0.14)	326 ^sh^, 340, 408 ^sh^, 425	510	-	-
	Film	325	-	390	1.1	-
	Blend PVK:PBD ^b^	310 ^sh^, 344 ^sh^	-	391, 481	2.1, 2.4	-
	Blend PVK:PBD ^c^	310 ^sh^, 344 ^sh^	-	373, 492	2.0, 1.8	-
AzNI-4	CHCl_3_	339 ^sh^, 389 ^sh^	328 ^sh^, 342, 409, 426 ^sh^	520	1.0	14.6
	CH_2_Cl_2_	330 (3.25), 330 ^sh^	327 ^sh^, 340, 407, 424 ^sh^	512	-	-
	CO(CH_3_)_2_	338 (2.76), 377 ^sh^	342, 411 ^sh^, 427	506	-	-
	CH_3_OH	326 (3.02), 376 ^sh^, 430 (0.11)	342, 406 ^sh^, 427	525	-	-
	CH_3_CN	327 (2.80), 378 ^sh^	324 ^sh^, 339, 410, 427 ^sh^	416, 492	-	-
	Film	338 ^sh^, 376 ^sh^, 397 ^sh^	-	-	-	-
	Blend PVK:PBD ^b^	310 ^sh^, 344 ^sh^	-	392, 465 ^sh^	1.3	-
	Blend PVK:PBD ^c^	310 ^sh^, 344 ^sh^	-	376, 493	0.8, 1.1	-
AzNI-5	CHCl_3_	341 (3.11), 375 ^sh^	328 ^sh^, 340, 409, 425 ^sh^	524	1.0	10.4
	CH_2_Cl_2_	337 (3.11), 377 ^sh^	326 ^sh^, 341, 409, 424 ^sh^	511	-	-
	CO(CH_3_)_2_	339 (3.00), 375 ^sh^	341, 410 ^sh^, 425	509	-	-
	CH_3_OH	335 (3.05), 380 ^sh^	342, 409 ^sh^, 428	548	-	-
	CH_3_CN	336 (3.20), 375 ^sh^	326 ^sh^, 340, 409 ^sh^, 425	507	-	-
	Film	354, 395 ^sh^, 423 ^sh^	^-^	-	-	-
	Blend PVK:PBD ^b^	310 ^sh^, 344 ^sh^	-	380, 469	1.2, 0.9	-
	Blend PVK:PBD ^c^	310 ^sh^, 344 ^sh^	-	377, 469	1.4, 1,1	-
AzNI-6	CHCl_3_	331 (3.30), 381 ^sh^	342, 408, 424 ^sh^	430, 514	3.2	11.5
	CH_2_Cl_2_	332 (3.38), 383 ^sh^	326 ^sh^, 340, 406, 424 ^sh^	432, 504	-	-
	CO(CH_3_)_2_	337 (3.15), 382 ^sh^	340, 410 ^sh^, 425	426, 500	-	-
	CH_3_OH	335 ^sh^, 330 ^sh^, 450 ^sh^	342, 410 ^sh^, 425	434, 536	-	-
	CH_3_CN	334 ^sh^, 379 ^sh^	324 ^sh^, 340, 412, 424	428, 502	-	-
	Film	328	-	504	1.2	-
	Blend PVK:PBD ^b^	310 ^sh^, 344 ^sh^	-	395, 468	3.6, 2.8	-
	Blend PVK:PBD ^c^	310 ^sh^, 344 ^sh^	-	380, 492	3.3, 4.2	-

c_solution_ = 10^−5^ mol/dm^3^, ^sh^ means shoulder, ^a^ Absorption coefficient, (dm^3^∙mol^−1^∙cm^−1^) ^b^ 2wt.% concentration of the compound in the PVK:PBD matrix (50:50 wt.%); ^c^ 15 wt.% concentration of the compound in the PVK:PBD matrix (50wt%:50wt%). The dominant band have been underlined.

**Table 4:** The position of the electroluminescence band maximum with its intensity.

		The Active Layer Construction		
Molecules	Parameters	/AzNI/	/PVK:PBD:AzNI 2 wt.%/	/PVK:PBD:AzNI 15 wt.%/
AzNI-1	λ_EL_ (nm)	-	497	565
	U_ELMax_ (V)	-	14	26
	EL intensity (counts)	-	7332	2425
AzNI-2	λ _EL_ (nm)	636	507	525
	U_ELMax_ (V)	16	27	20
	EL intensity (counts)	6390	17410	19,602
AzNI-3	λ _EL_ (nm)	-	504	507
	U_ELMax_ (V)	-	25	17
	EL intensity (counts)	-	62,103	25,123
AzNI-4	λ _EL_ (nm)	-	495	525
	U_ELMax_ (V)	-	17	25
	EL intensity (counts)	-	44,236	3058
AzNI-5	λ _EL_ (nm)	-	503	-
	U_ELMax_ (V)	-	26	-
	EL intensity (counts)	-	8956	-
AzNI-6	λ _EL_ (nm)	-	500	536
	U_ELMax_ (V)	-	25	24
	EL intensity (counts)	-	39205	1280

λ_EL_—the maximum of the electroluminescence band. U_ELMax_—external voltage for maximal electroluminescence intensity.

**Table 5:** Cytotoxicity of the tested compounds on a panel of three human cancer cell lines and normal human fibroblasts.

Molecules	Activity [µM]			
	MCF-7	HCT 116	U-87	NHDF
AzNI-1	>25	>25	>25	>25
AzNI-2	>25	>25	>25	>25
AzNI-3	>25	>25	>25	>25
AzNI-4	>25	>25	>25	>25
AzNI-5	>25	>25	>25	>25
AzNI-6	>25	>25	>25	>25

## Data Availability

Data are contained within the article and supplementary material.

## Supplementary Materials

The following are available online at https://www.mdpi.com/article/10.3390/ma14195504/s1, Figure S1: ^1^HNMR of the investigated compounds (400 MHz, DMSO-d_6_). Figure S2: COSY and HMQC of AzNI-1 (400 MHz, DMSO-d_6_). Figure S3: TGA thermograms on the left and DTG thermograms of the right. Figure S4: DSC thermogram of the melting temperature in the first heating scan of AzNI-1. Figure S5: The (a) absorption, (b) excitation and (c) emission spectra of N-hexyl-1,8-naphthalimides derivatives (AzNI-1,3,4,5,6) in various solvents. Figure S6: The excitation and absorption spectra of the N-hexyl-1,8-naphthalimides derivatives (AzNI-1–6) in the chloroform and thin films with the emission spectra of PVK:PBD matrix. Figure S7: The absorption, excitation and PL spectra’s win selected media of AzNI-1, AzNI-2 and AzNI-6. Figure S8: The 3D fluorescence spectra of amine in the excitation range from 320 to 500 nm and the collected emissions in the range from 350 to 650 nm in the in chloroform (CHCl_3_), acetonitryle (CH_3_CN) and methanol (CH_3_OH). Measurements were performed for concentration of amine c = 1·10^−5^ mol/dm^3^ and under the same measurement conditions. Figure S9: Effect of TFA on photoluminescence (PL) properties of Amine in chloroform (CHCl_3_), acetonitryle (CH_3_CN) and methanol (CH_3_OH): (a) superimposed spectra at excitation 340 or 430 nm, (b) λ_em_ intensity versus equivalent of TFA. Photographs were taken under 366 nm UV irradiation from a hand-held UV lamp. Figure S10: The 3D fluorescence spectra of analyzed compounds in the excitation range from 320 to 500 nm and the collected emissions in the range from 350 to 650 nm in the chloroform (CHCl_3_), acetonitryle (CH_3_CN) and methanol (CH_3_OH). Measurements were performed for equal concentration of each compound (c = 1·10^−5^ mol/dm^3^) and under the same measurement conditions. Figure S11: Photoluminescence (PL) properties of imines (AzNI-1,3,4,5,6) in a binary mixture of MeOH/H_2_O: (a) with an increasing water content (*fw*) at excitation of 340 and 430 nm, (b) λ_em_ intensity versus the water content (*fw*) in the solvents mixture. Photographs were taken under 366 nm UV irradiation from a hand-held UV lamp. Figure S12: Effect of TFA on photoluminescence (PL) properties of imines (AzNI-1,3,4,5) in chloroform (CHCl_3_): (a) superimposed spectra at excitation 340 or 430 nm, (b) λ_em_ intensity versus equivalent of TFA. Photographs were taken under 366 nm UV irradiation from a hand-held UV lamp. Figure S13: Effect of TFA on photoluminescence (PL) properties of imines (AzNI-1,3,4,5) in acetonitryle (CH_3_CN): (a) superimposed spectra at excitation 340 or 430 nm, (b) λ_em_ intensity versus equivalent of TFA. Photographs were taken under 366 nm UV irradiation from a hand-held UV lamp. Figure S14: Effect of TFA on photoluminescence (PL) properties of imines (AzNI-1,3,4,5) in mathanol (CH_3_OH): (a) superimposed spectra at excitation 340 or 430 nm, (b) λ_em_ intensity versus equivalent of TFA. Photographs were taken under 366 nm UV irradiation from a hand-held UV lamp. Figure S15: Effect of TFA on photoluminescence (PL) properties of imine AzNI-6 in chloroform (CHCl_3_), acetonitryle (CH_3_CN) and methanol (CH_3_OH): (a) superimposed spectra at excitation 340 or 430 nm, (b) λ_em_ intensity versus equivalent of TFA. Photographs were taken under 366 nm UV irradiation from a hand-held UV lamp. Figure S16: Emission spectra for the tested compounds in chloroform at various concentrations (from 100 to 1 μM) at excitation 340 nm (black line) and 430 nm (red line). Measurements were performed for the same measurement conditions. Figure S17: Excitation and emission spectra for the tested compounds in methanol at various concentrations (100 and 10 μM). Measurements were performed for the same measurement conditions. Figure S18: Co-localization fluorescence images of AzNI-3 at a concentration of 25 µM and ERTracker Red dye in MCF-7 cells. The images acquired under 470 nm (for AzNI-3) and 587 nm (for ER-dye) LED illumination. Scale bars indicate 25 µm.
