# Supplementary material for: Luminescence and Electrochemical Activity of New Unsymmetrical 3-Imino-1,8-naphthalimide Derivatives
Source: Materials (Basel). 2021 Sep 23;14(19):5504. doi: 10.3390/ma14195504 (PMC8509721; doi:10.3390/ma14195504)
Supplement: Supplementary file 1 [file materials-14-05504-s001.zip › materials-1366538-supplementary.pdf]

Luminescence and electrochemical activity of new unsymmetrical 3-imino-1,8-naphthalimide derivatives

Sonia Kotowicz[1*], Mateusz Korzec[*1], Katarzyna Malarz[2], Aleksandra Krystkowska[2], Anna Mrozek-Wilczkiewicz[2], Sylwia Golba[3], Mariola Siwy[4], Sebastian Maćkowski[5] and Ewa Schab-Balcerzak[1,4]

[1]Institute of Chemistry, University of Silesia, 9 Szkolna Str., 40-006 Katowice, Poland
[2]A. Chelkowski Institute of Physics, University of Silesia in Katowice,1A 75 Pulku Piechoty Str., 41-500 Chorzow, Poland
[3]Institute of Materials Science, University of Silesia,1A 75 Pulku Piechoty Str., 41-500 Chorzow, Poland
[4]Centre of Polymer and Carbon Materials, Polish Academy of Sciences, 34 M. Curie-Sklodowska Str., 41-819 Zabrze, Poland
[5]Institute of Physics, Faculty of Physics, Astronomy and Informatics, Nicolaus Copernicus University, 5 Grudziadzka Str., 87-100 Torun, Poland
* corresponding authors: sonia.kotowicz@us.edu.pl, mateusz.korzec@us.edu.pl

1. Materials and characterization methods

β-Phenylcinnamaldehyde, 9-phenanthrenecarboxaldehyde, 4-bromobenzaldehyde, 2,4-dichlorobenzaldehyde, p-nitrobenzaldehyde, 4-(1*H*-imidazol-1-yl)benzaldehyde, Pd/C, 3-nitro-1,8-naphthalic anhydride, hydrazine 98%, hexylamine, poly(*N*-vinylcarbazole) (PVK) and 2-(4-tert-butylphenyl)-5-(4-biphenylyl)-1,3,4-oxadiazole (PBD), Bu_4NPF_6, dichloromethane (DCM) and other solvents were purchased from Sigma Aldrich (Merck). Poly(3,4(ethylenedioxy)thiophene):poly(styrenesulfonate) (PEDOT:PSS) and ITO substrates glass were purchased from OSSILA.

2. Blends and films preparation

Films and blends on the glass substrates were prepared from a homogeneous chloroform solution (10 mg/ml) of core substituted *N*-hexyl-1,8-naphthalimides (and with PVK:PBD (50:50 in weight %) (2 or 15 wt. % AzNIs content), respectively, by spin-coating (1000 rpm, 60s; Laurell WS 650MZ-23NPPB). Then films and blends were dried for 24h in a vacuum oven (BUCHI Glass oven B-585) at 60°C.

3. OLED preparation and electroluminescence measurements

Devices with sandwich configuration ITO/PEDOT:PSS/**AzNI**/Al and ITO/PEDOT:PSS/**AzNI**:PVK:PBD/Al with 2 and 15 wt. % of **AzNIs** content in blend were prepared. Devices were prepared on OSSILA substrates with pixilated ITO anodes, cleaned with detergent, deionized water, 10% NaOH solution, water and isopropyl alcohol in an ultrasonic bath. Substrates were covered with PEDOT:PSS film by spin coating at 5000rpm for 60s and annealed for 5 min at 120°C. Active layer was spin-coated on top of the PEDOT:PSS layer from chloroform solution (10 mg/mL) at 1000 rpm for 60 s and annealed for 5 min at 100°C. Finally Al was vacuum-deposited at a pressure of $5{\cdot}10^{-5}$ Torr. Electroluminescence (EL) spectra were measured with the voltage applied using a precise voltage supply (GwInstek PSP-405) and the sample was fixed to an XYZ stage. Light from the OLED device was collected through a 30mm lens, focused on the entrance slit (50μm) of a

monochromator (Shamrock SR-303i) and detected using a CCD detector (AndoriDus 12305). Typical acquisition times were equal to 10 seconds. The pre-alignment of the setup was done using a 405 nm laser. The layers thickness were measured using an AFM Park XE-100 instrument (Park System, Korea) operating in tapping mode in the air with standard 125 μm single-crystal silicon cantilevers. The piezoelectric scanner had a scan range of approximately 100 μm x 100 μm.

4. Characterization Methods

The synthesis were performed using IKA C-MAG HS7 (IKA® Poland Sp. z o.o., Warszawa, Poland) and VWR VMS-C7 (WR International, LLC.) hot plates. Nuclear magnetic resonance (^{1}H and ^{13}C NMR) spectra were recorded on a Bruker AC400 spectrometer (Berlin, Germany) in DMSO-d_6 as solvent and TMS as the internal standard. Infrared spectra (FTIR) were recorded on a Thermo Scientific Nicolet iS5 FT-IR Spectrometer in the range of 4000 - 400 cm^{-1} as KBr pressed pellets (KBr before use was dried). The elementary analysis was performed using Vario EL III apparatus (Elementar, Langenselbold, Germany). Differential Scanning Calorimetry (DSC) was performed using a Du Pont 1090B apparatus (US) with a heating/cooling rate of 20 ºC·min^{-1} under nitrogen and using aluminum sample pans in the range of 0 – 250°C. The glass transition temperature was recorded in the second scan after cooling. Thermogravimetric analysis (TGA) was done with a Mettler Toledo TGA STARe system (Warszawa, Poland) with a heating rate of 10 °C·min^{-1} in a constant stream of nitrogen (20 ml·min^{-1}) and a temperature range from 35 °C to 500 °C. UV-Vis absorption spectra were performed using an Evolution 220 UV-Visible Spectrophotometer (London, United Kingdom) as the concentration of 10^{-5} mol/L and 1 cm quartz cell and Jasco V-550 Spectrophotometer (Easton, MD 21601) for films and blends. Photoluminescence spectra (PL) in solutions were performed by using Varian Carry Eclipse Spectrometer (Santa Clara, US) and using Hitachi F-2500 Spectrometer (Berkshire, United Kingdom) for blends and films PL measurements. Quantum yields (Φ_f) measurements were performed by using the integrating sphere Avantes AvaSphere-80 (Edinburgh Instruments, Austion TX, US) and absolute method. The lifetime (τ) of photoluminescence was measured with a time-correlated single photon counting (TCSPC). The time-resolved measurements were performed using the picosecond pulsed diode laser, EPL-340 nmand using a 60 W microsecond Xe flash lamp. Pulse period for all measurements was equal to 50 ns and PMT (Hamamatsu, R928P) as a detector. The fluorescence decay analysis was received an instrument response function (IRF) using ludox solution and results were presented as average values of decay after exponential fitting. Electrochemical measurements (CV) were performed with Eco ChemieAutolab PGSTAT128n potentiostat (Metrohm Autolab B.V. The Netherlands). A platinum wire (diam. 2.0 mm) served as a working electrode, while platinum coil and silver wire were used as auxiliary and reference electrode respectively. All potentials were referenced with respect to the stable internal standard, which was ferrocene couple (Fc/Fc^+). Electrochemical experiments were conducted in a one-compartment cell, in CH_2Cl_2 (ACROSS Organics, 99.9% for

biochemistry grade), under argon purging, with 0.1M Bu_4NPF_6 (Aldrich, 99%) used as the supporting electrolyte salt. Each experiment was performed in an air-conditioned room in stable temperature (t = 20 °C). The measurements were recorded with moderate scan rate equal to 0.10 V/s for CV and 0.05 for DPV.

5. NMR spectra

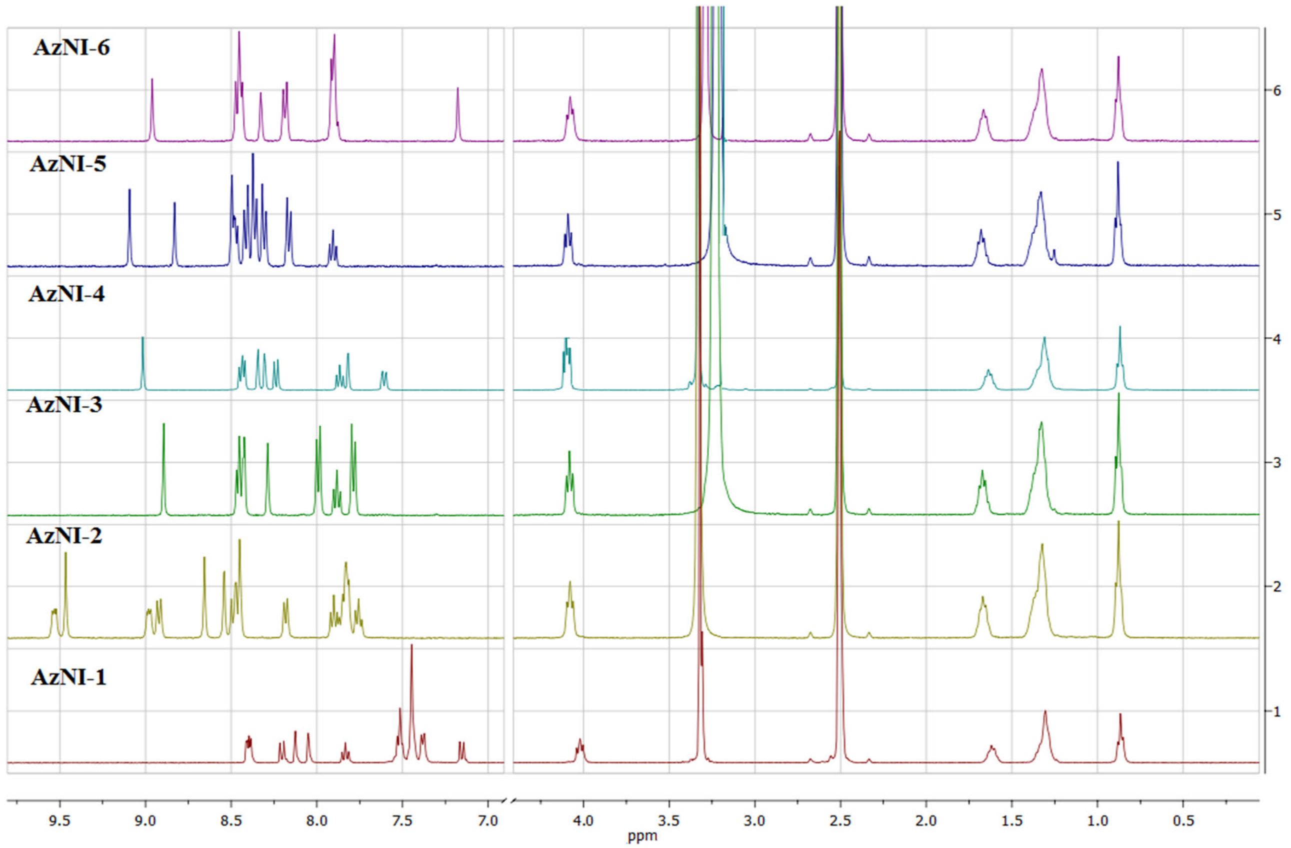

Figure S1. ^{1}HNMR of the investigated compounds (400 MHz, DMSO-d_6).

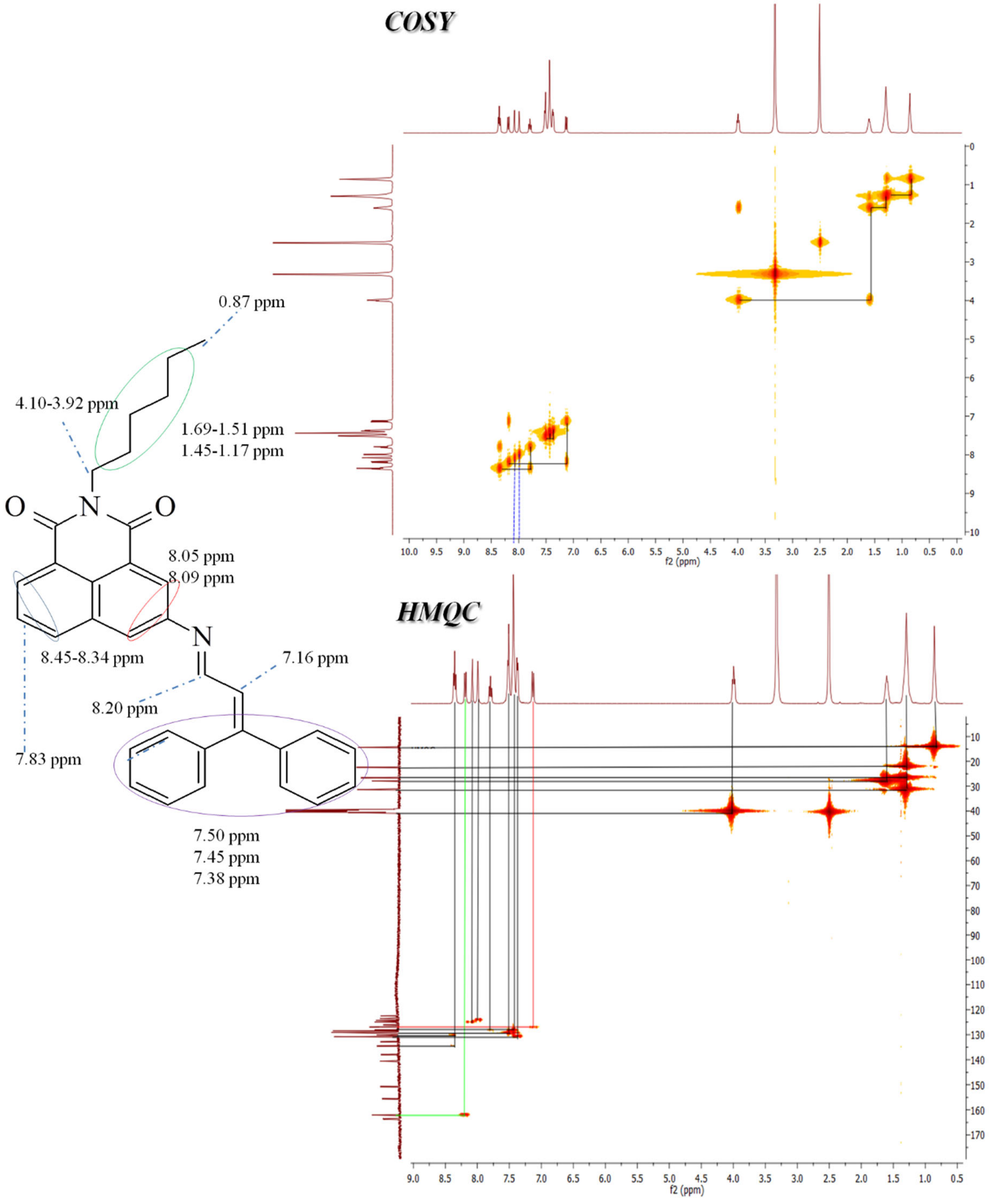

Figure S2. COSY and HMQC of **AzNI-1** (400 MHz, DMSO-d_6).

6. Thermal investigations.

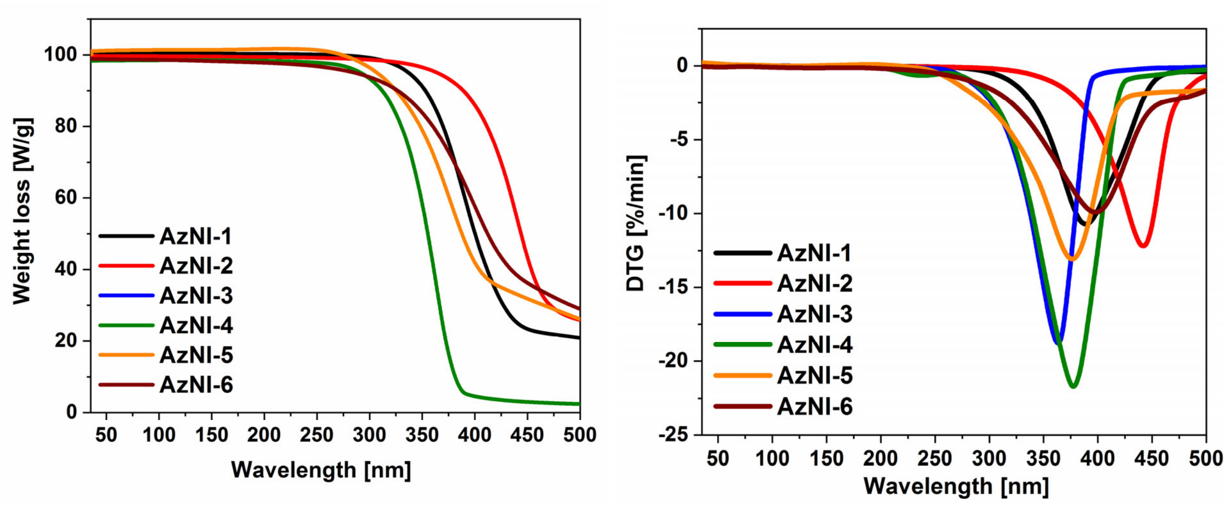

Figure S3. TGA thermograms on the left and DTG thermograms of the right.

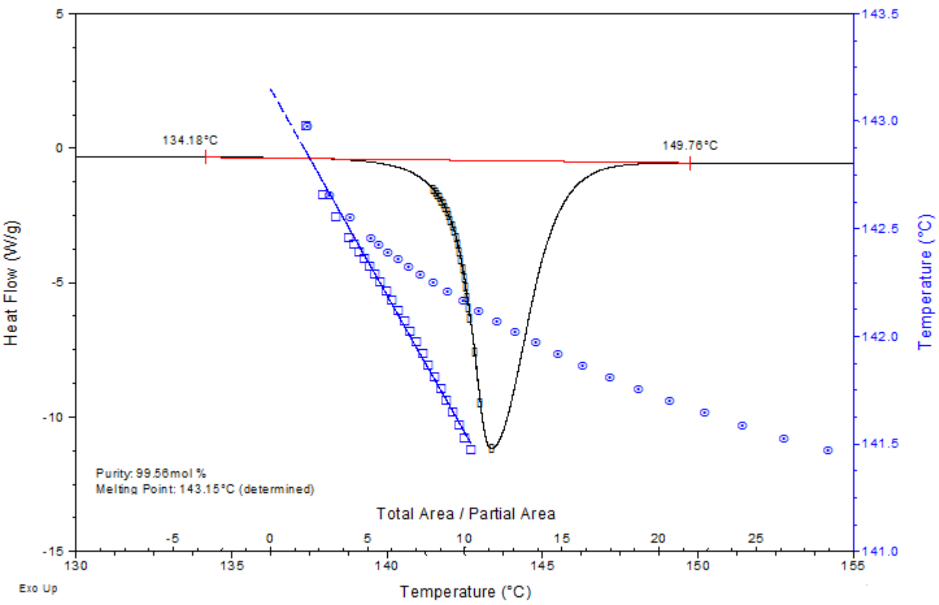

Figure S4. DSC thermogram of the melting temperature in the first heating scan of **AzNI-1**.

7. Optical properties

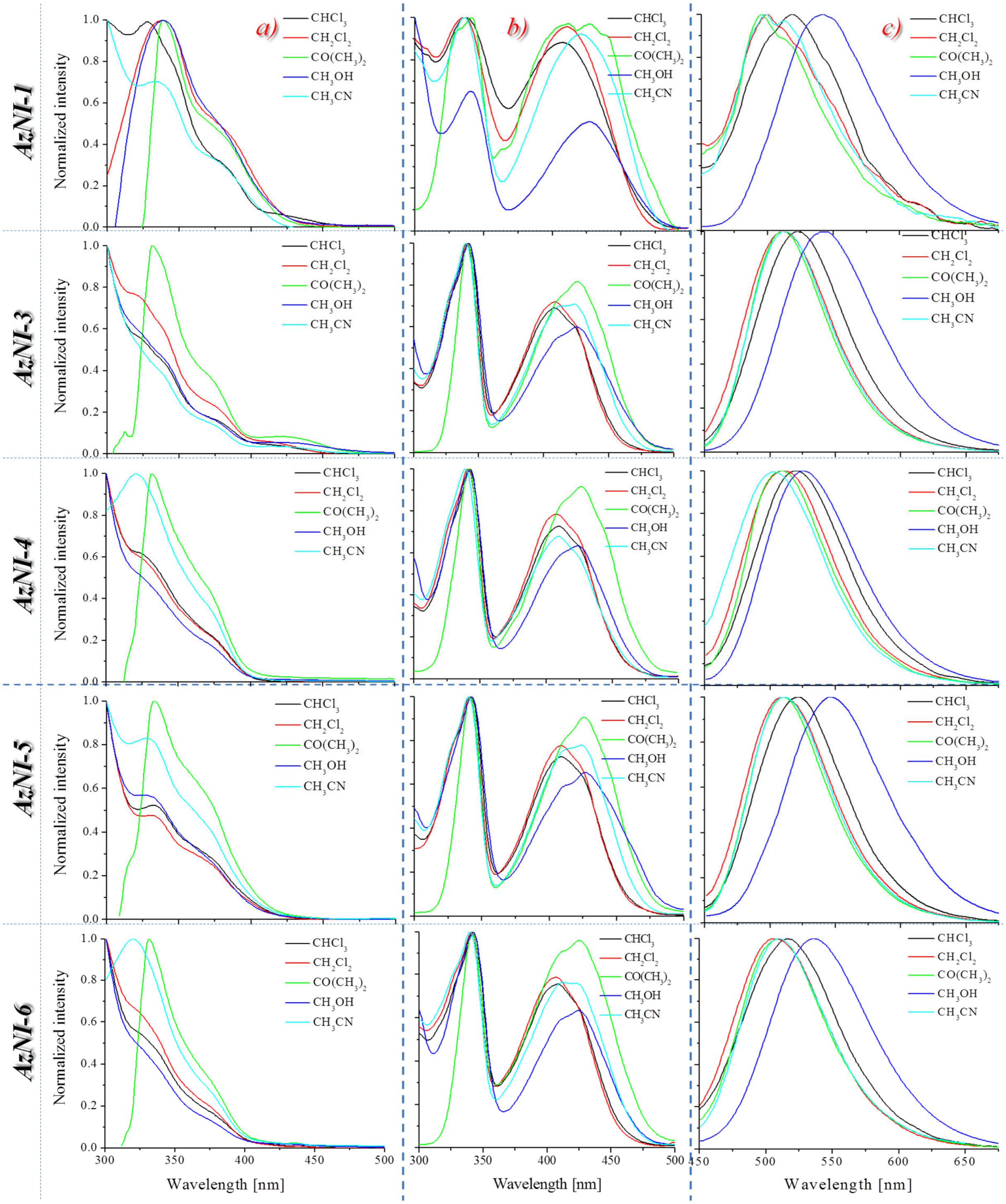

Figure S5. The (a) absorption, (b) excitation and (c) emission spectra of *N*-hexyl-1,8-naphthalimides derivatives (**AzNI- 1**, **3, 4, 5, 6**) in various solvents.

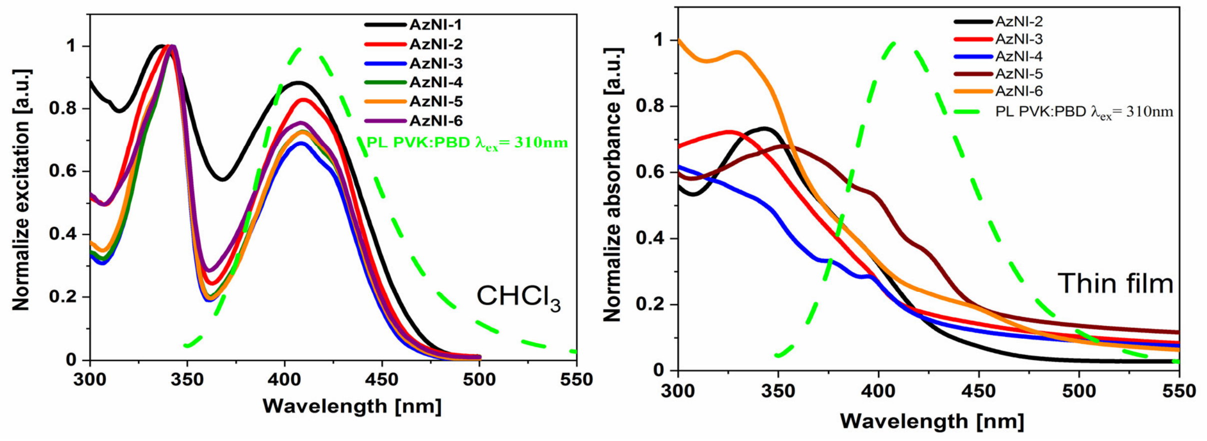

Figure S6. The excitation and absorption spectra of the *N*-hexyl-1,8-naphthalimides derivatives (**AzNI-1** - **6**) in the chloroform and thin films with the emission spectra of PVK:PBD matrix.

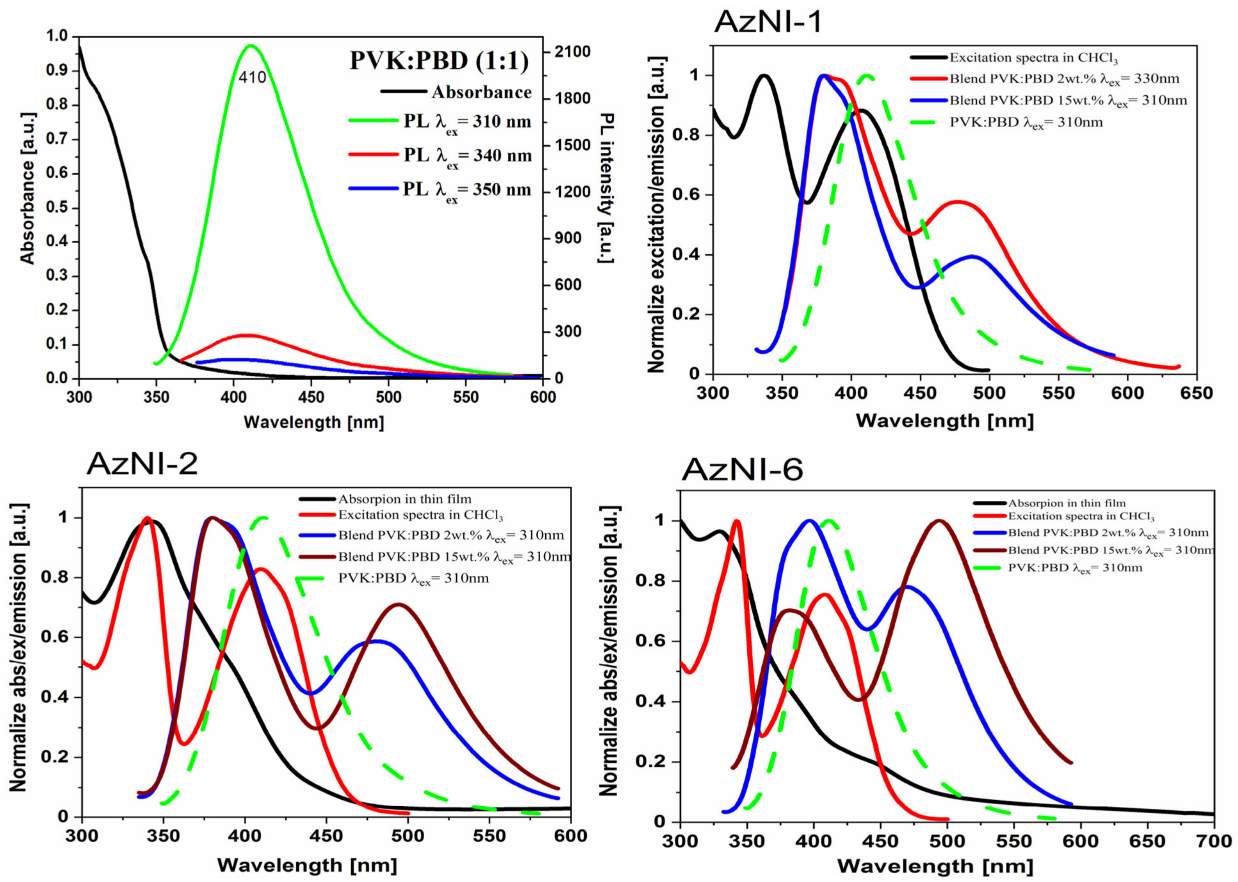

Figure S7. The absorption, excitation and PL spectra's win selected media of **AzNI-1**, **AzNI-2** and **AzNI-6**.

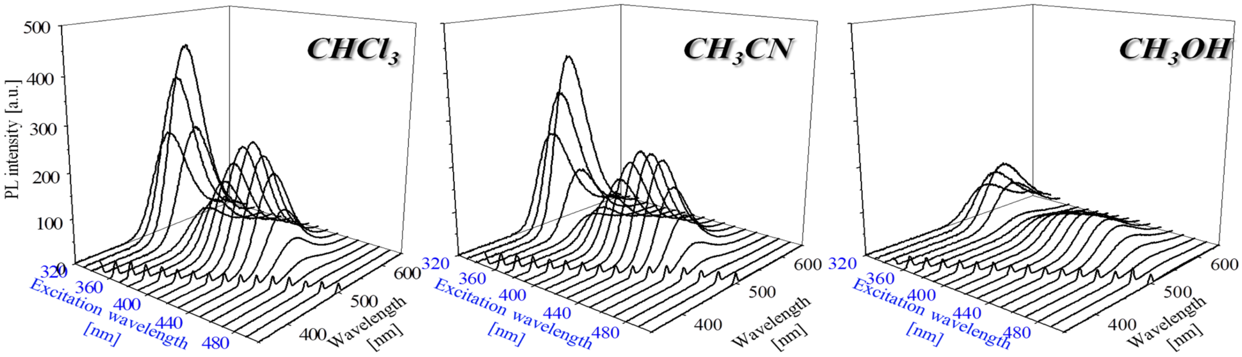

Figure S8. The 3D fluorescence spectra of amine in the excitation range from 320 to 500 nm and the collected emissions in the range from 350 to 650 nm in the in chloroform ($CHCl_3$), acetonitryle (CH_3CN) and methanol (CH_3OH). Measurements were performed for concentration of amine c = $1{\cdot}10^{-5}$ mol/dm^3 and under the same measurement conditions.

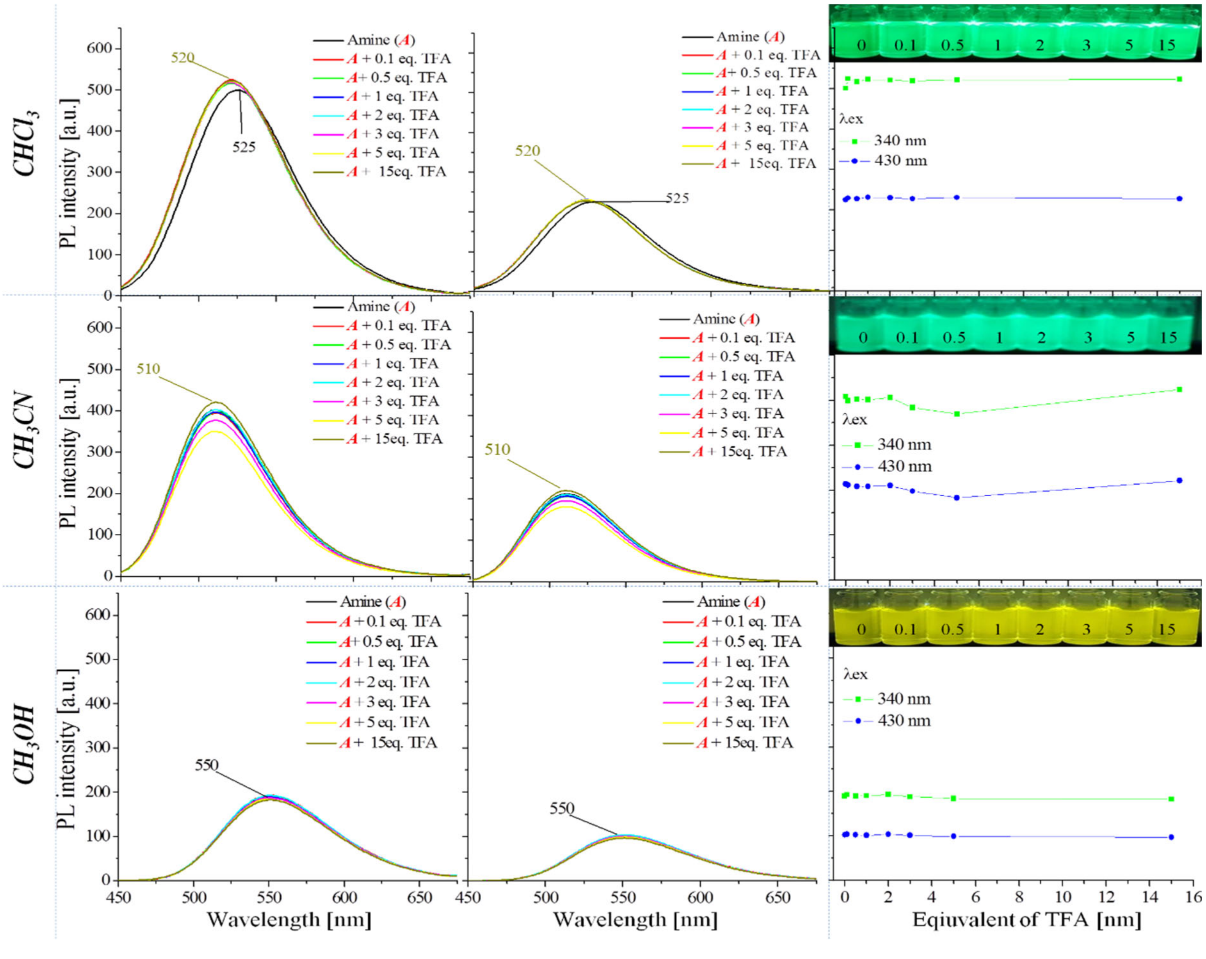

Figure S9. Effect of TFA on photoluminescence (PL) properties of Amine in chloroform ($CHCl_3$), acetonitryle (CH_3CN) and methanol (CH_3OH): a) superimposed spectra at excitation 340 or 430 nm, (b) λ_{em} intensity versus equivalent of TFA. Photographs were taken under 366 nm UV irradiation from a hand-held UV lamp.

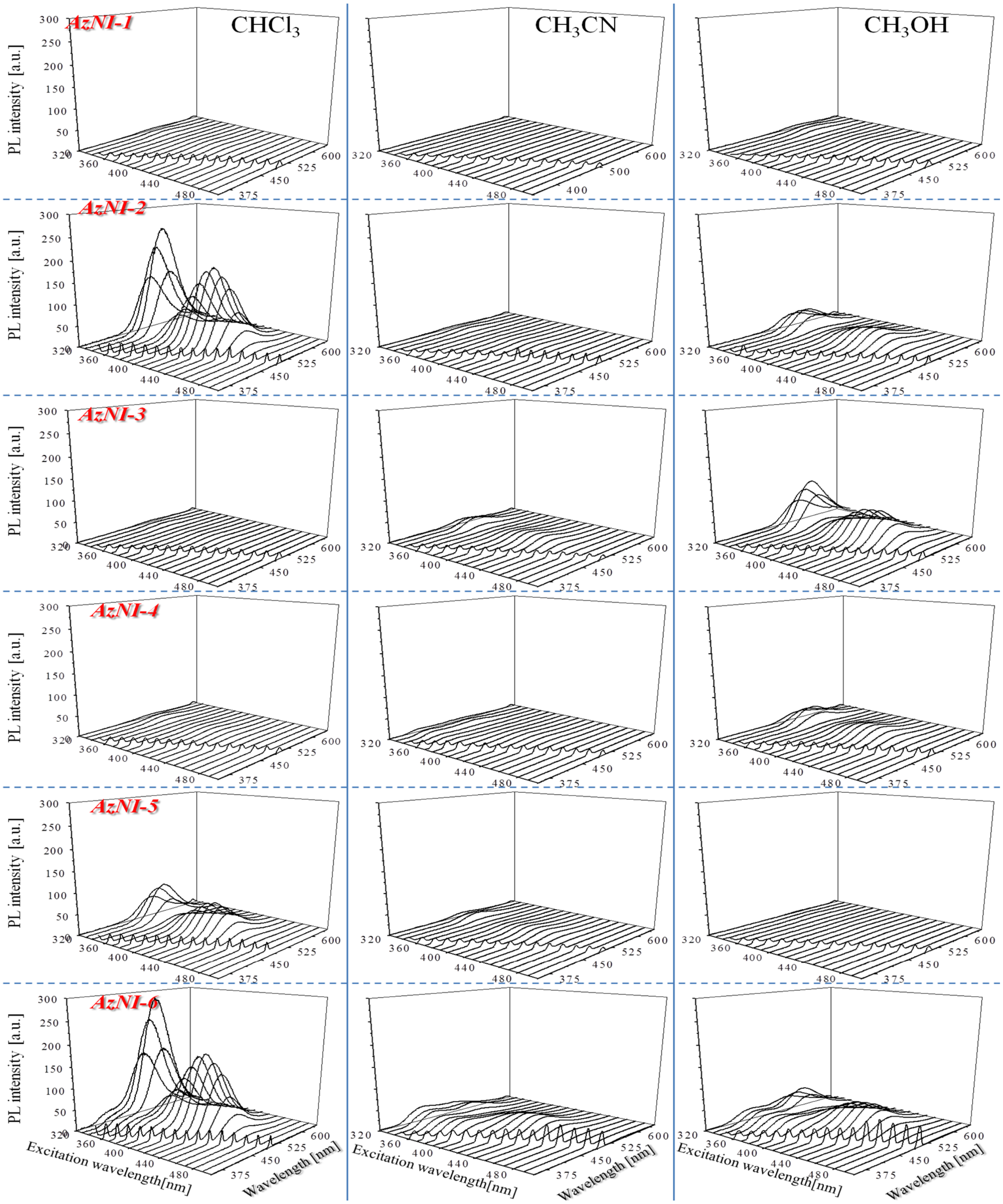

Figure S10. The 3D fluorescence spectra of analyzed compounds in the excitation range from 320 to 500 nm and the collected emissions in the range from 350 to 650 nm in the chloroform ($CHCl_3$), acetonitryle (CH_3CN) and methanol (CH_3OH). Measurements were performed for equal concentration of each compound (c = 1·10^{-5} mol/dm^3) and under the same measurement conditions.

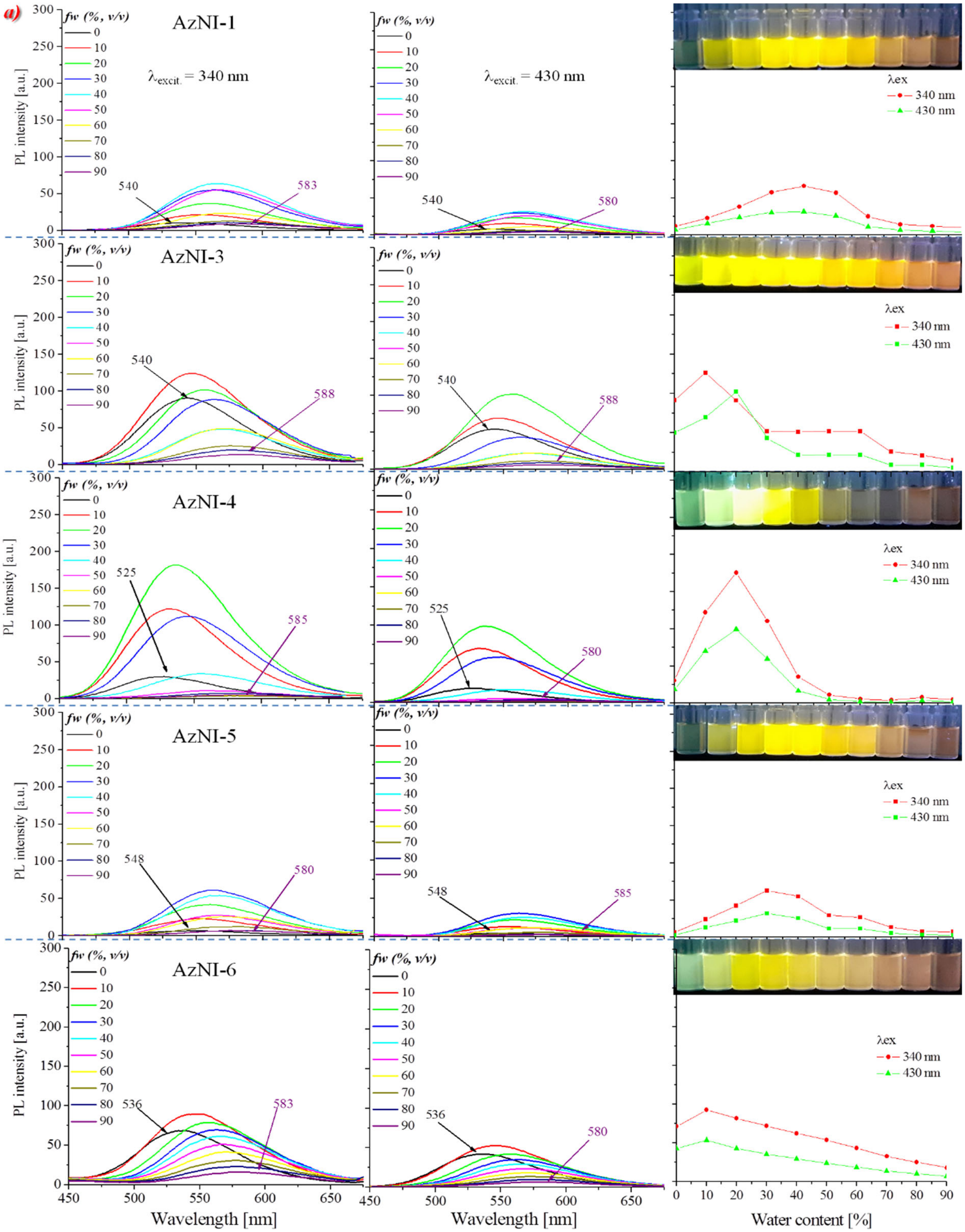

Figure S11. Photoluminescence (PL) properties of imines (**AzNI- 1, 3, 4, 5, 6**) in a binary mixture of MeOH/H_2O: a) with an increasing water content (*fw*) at excitation of 340 and 430 nm, (b) λ_{em} intensity versus the water content (*fw*) in the solvents mixture. Photographs were taken under 366 nm UV irradiation from a hand-held UV lamp.

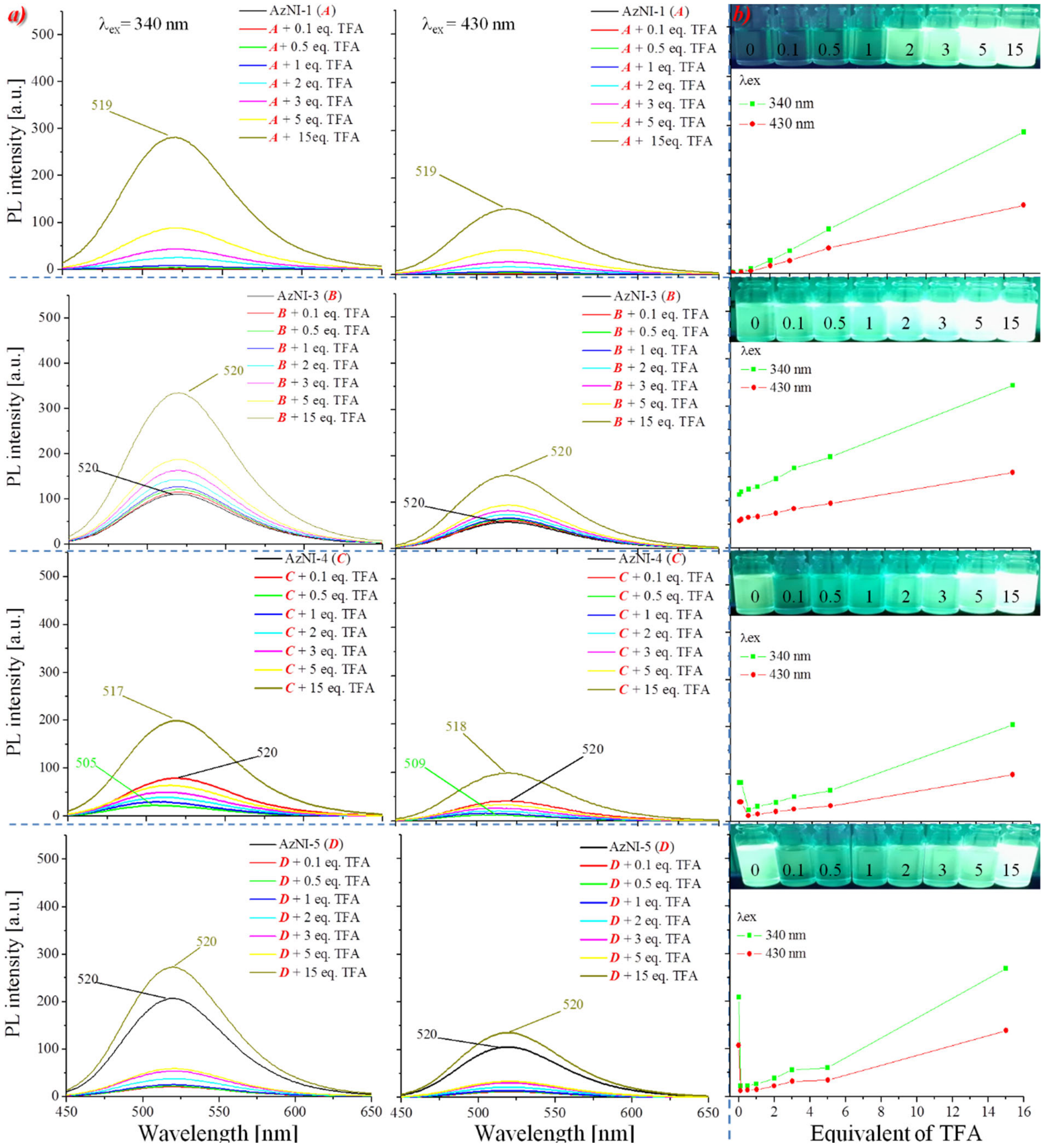

Figure S12. Effect of TFA on photoluminescence (PL) properties of imines (**AzNI-1,3,4,5**) in chloroform ($CHCl_3$): a) superimposed spectra at excitation 340 or 430 nm, (b) λ_{em} intensity versus equivalent of TFA. Photographs were taken under 366 nm UV irradiation from a hand-held UV lamp.

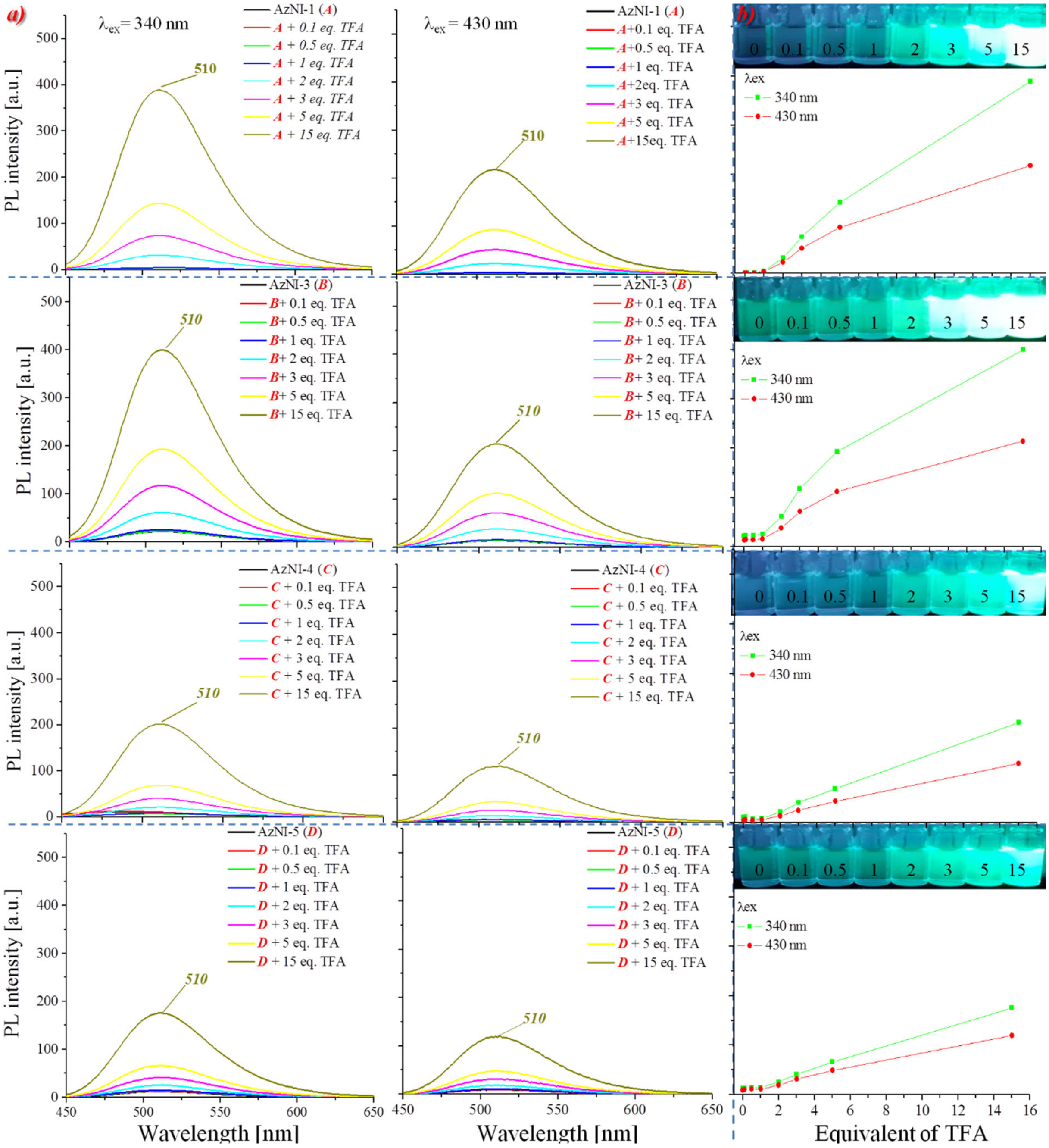

Figure S13. Effect of TFA on photoluminescence (PL) properties of imines (**AzNI- 1,3,4,5**) in acetonitryle (CH_3CN): a) superimposed spectra at excitation 340 or 430 nm, (b) λ_{em} intensity versus equivalent of TFA. Photographs were taken under 366 nm UV irradiation from a hand-held UV lamp.

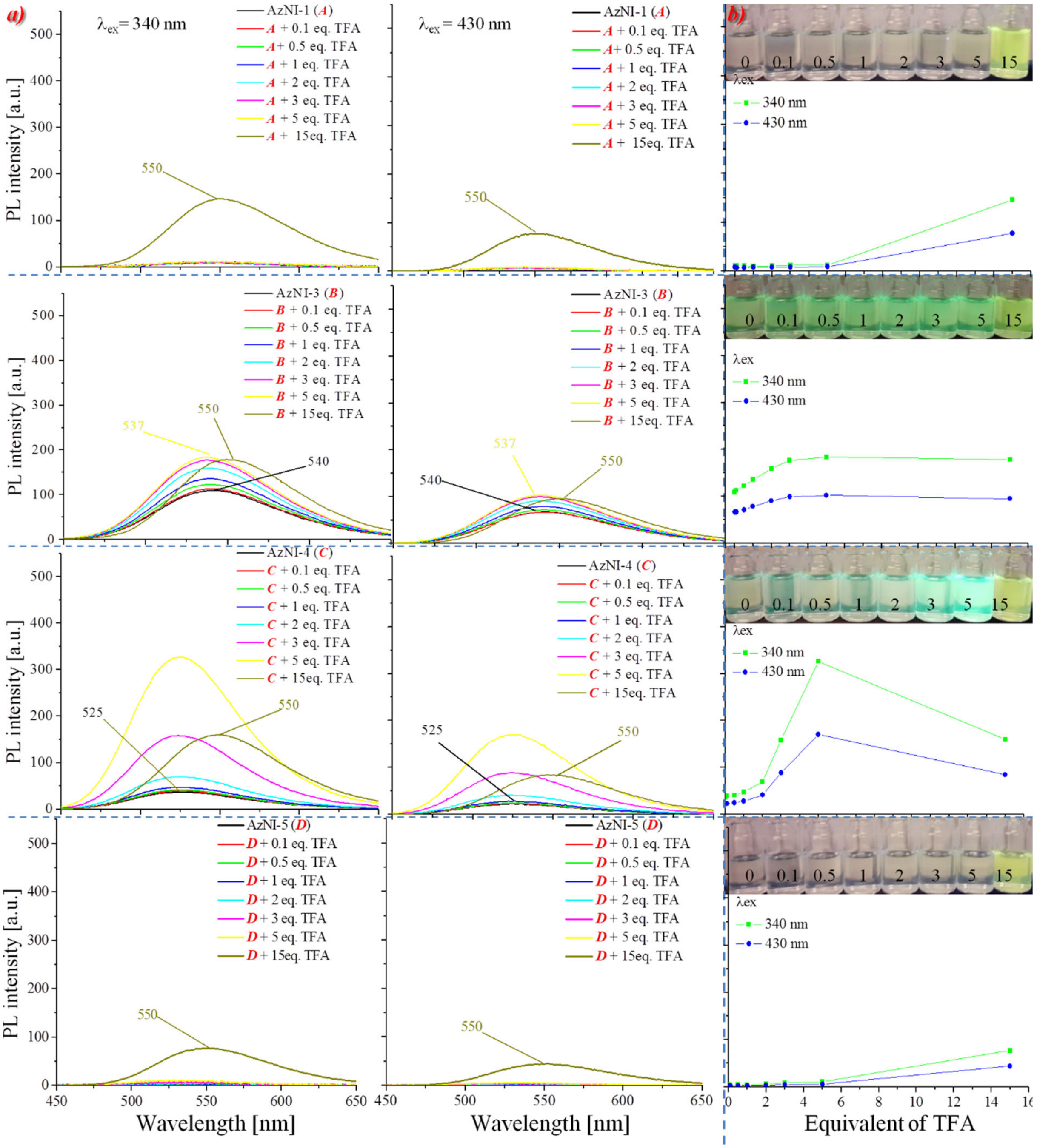

Figure S14. Effect of TFA on photoluminescence (PL) properties of imines (**AzNI 1,3,4,5**) in mathanol (CH_3OH): a) superimposed spectra at excitation 340 or 430 nm, (b) λ_{em} intensity versus equivalent of TFA. Photographs were taken under 366 nm UV irradiation from a hand-held UV lamp.

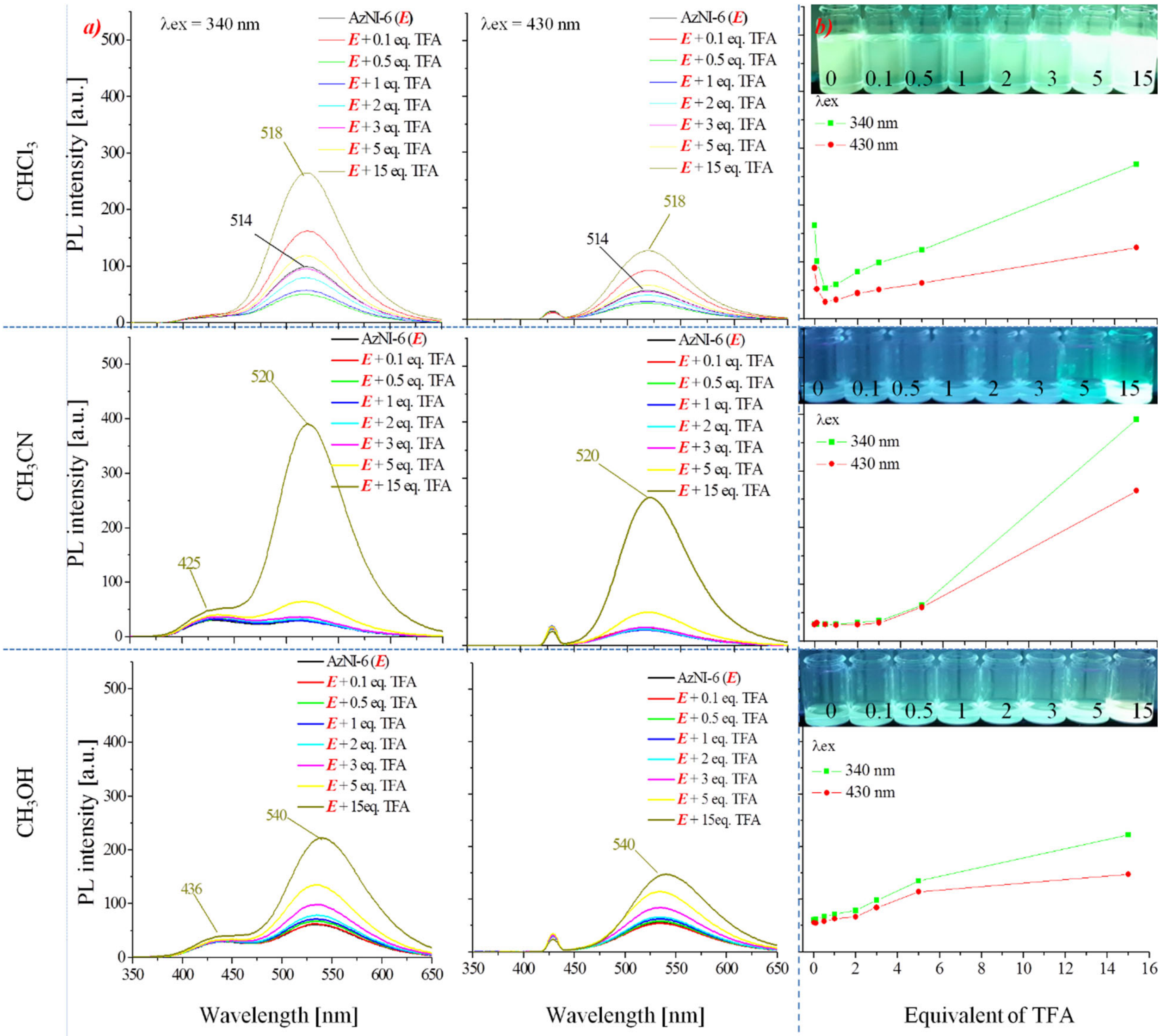

Figure S15. Effect of TFA on photoluminescence (PL) properties of imine **AzNI-6** in chloroform ($CHCl_3$), acetonitryle (CH_3CN) and methanol (CH_3OH): a) superimposed spectra at excitation 340 or 430 nm, (b) λ_{em} intensity versus equivalent of TFA. Photographs were taken under 366 nm UV irradiation from a hand-held UV lamp.

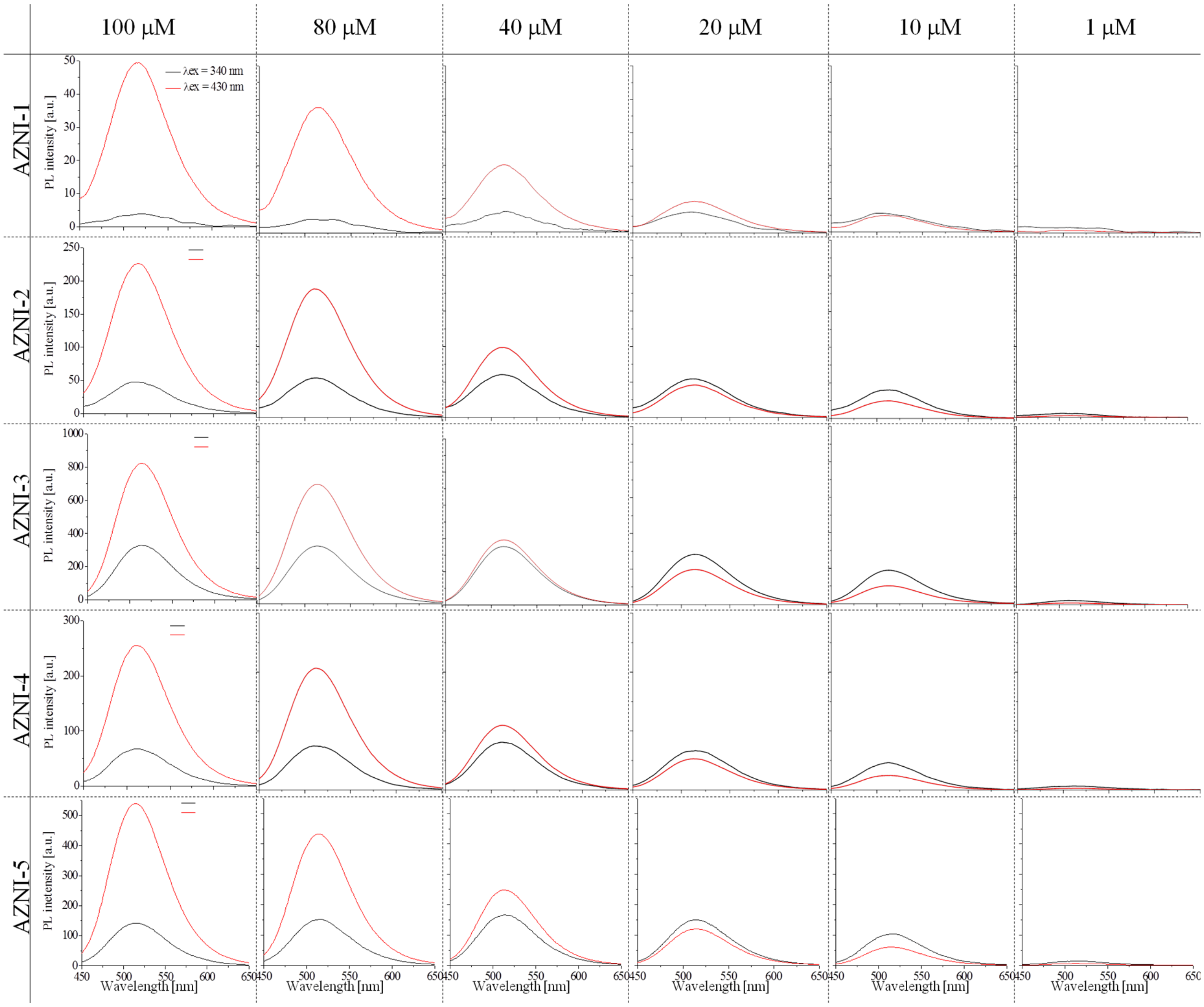

Figure S16. Emission spectra for the tested compounds in chloroform at various concentrations (from 100 to 1 µM) at excitation 340 nm (*black line*) and 430 nm (*red line*). Measurements were performed for the same measurement conditions.

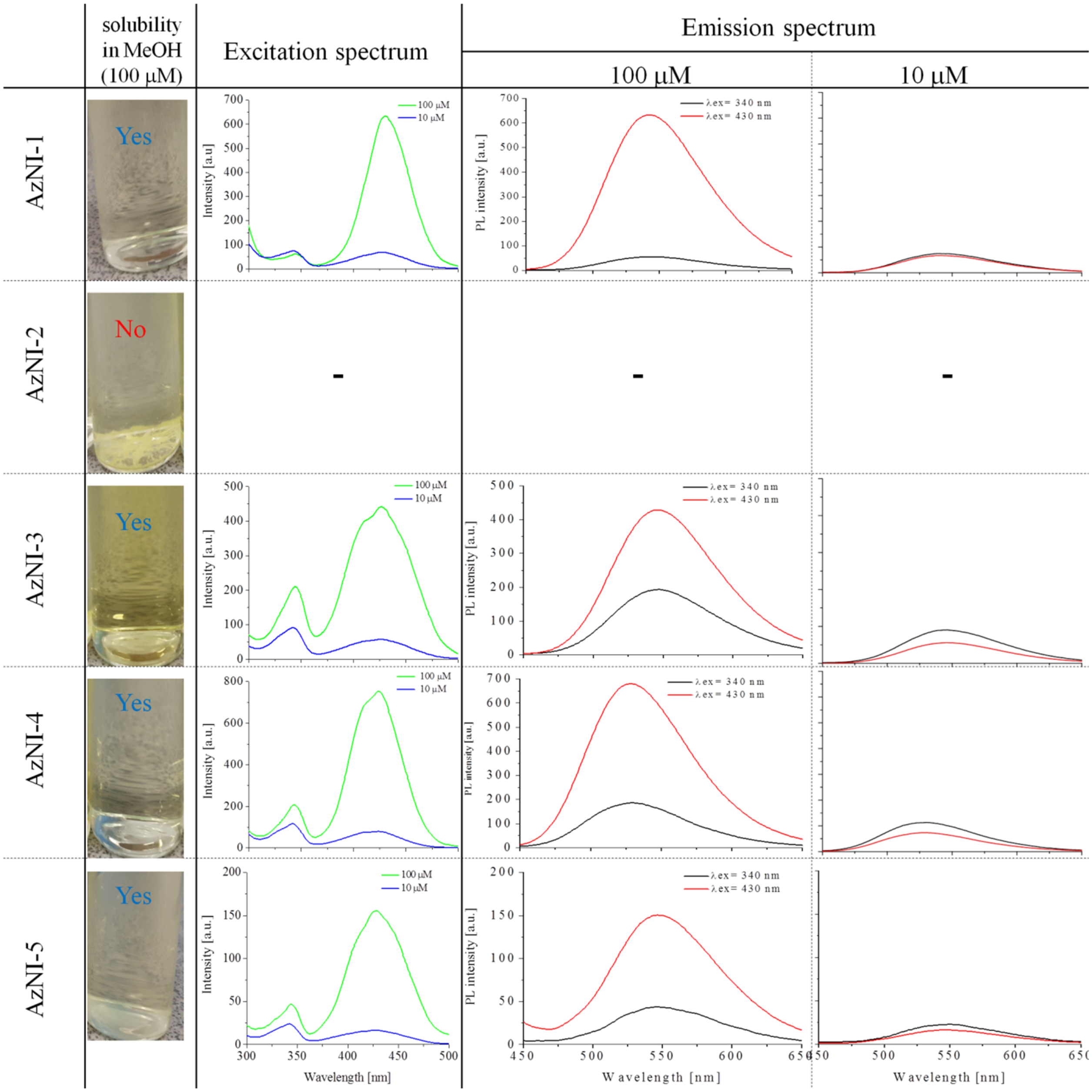

Figure S17. Excitation and emission spectra for the tested compounds in methanol at various concentrations (100 and 10 μM). Measurements were performed for the same measurement conditions.

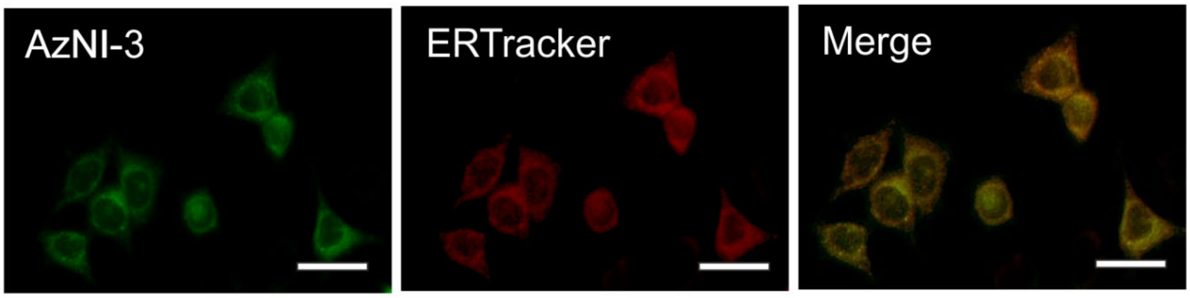

Figure S18. Co-localization fluorescence images of **AzNI-3** at a concentration of 25 μM and ERTracker Red dye in MCF-7 cells. The images acquired under 470 nm (for AzNI-3) and 587 nm (for ER-dye) LED illumination. Scale bars indicate 25 μm.
